# Detecting and Mitigating Attacks on GPS Devices

**DOI:** 10.3390/s24175529

**Published:** 2024-08-26

**Authors:** Jack Burbank, Trevor Greene, Naima Kaabouch

**Affiliations:** Artificial Intelligence Research (AIR) Center, University of North Dakota, Grand Forks, ND 58202, USA; trevor.greene@und.edu

**Keywords:** global positioning system, GPS security, GPS jamming, GPS spoofing, GPS-denied, environments

## Abstract

Modern systems and devices, including unmanned aerial systems (UASs), autonomous vehicles, and other unmanned and autonomous systems, commonly rely on the Global Positioning System (GPS) for positioning, navigation, and timing (PNT). Cellular mobile devices rely on GPS for PNT and location-based services. Many of these systems cannot function correctly without GPS; however, GPS signals are susceptible to a wide variety of signal-related disruptions and cyberattacks. GPS threat detection and mitigation have received significant attention recently. There are many surveys and systematic reviews in the literature related to GPS security; however, many existing reviews only briefly discuss GPS security within a larger discussion of cybersecurity. Other reviews focus on niche topics related to GPS security. There are no existing comprehensive reviews of GPS security issues in the literature. This paper fills that gap by providing a comprehensive treatment of GPS security, with an emphasis on UAS applications. This paper provides an overview of the threats to GPS and the state-of-the-art techniques for attack detection and countermeasures. Detection and mitigation approaches are categorized, and the strengths and weaknesses of existing approaches are identified. This paper also provides a comprehensive overview of the state-of-the-art on alternative positioning and navigation techniques in GPS-disrupted environments, discussing the strengths and weaknesses of existing approaches. Finally, this paper identifies gaps in existing research and future research directions.

## 1. Introduction

Modern systems and devices commonly rely on the Global Positioning System (GPS) for positioning, navigation, and timing (PNT). Manned and unmanned airplanes, ships, and ground vehicles typically rely on GPS for positioning and navigation. Unmanned autonomous systems, such as unmanned aerial systems (UASs), rely on GPS to support navigation. Cellular mobile devices utilize these navigation signals for self-positioning and supporting location-based services and applications.

Reliable and accurate positioning and navigation are paramount for safe and reliable UAS operations. A UAS must typically maintain an accurate record of where it has been, where it currently is, and where it is going to successfully complete its task. An unmanned sensor often needs to know its location for its measurements to be useful. A UAS must be capable of conducting accurate navigation to maintain flight safety. Autonomous unmanned operations are not possible without reliable positioning and navigation capabilities. A common approach to providing reliable UAS positioning and navigation is through the use of a Global Navigation Satellite System (GNSS), such as a GPS, which provides a series of signals from space-based satellites that ground or air-based systems use to determine their location and typically serves as a primary input into the system’s guidance and navigation system. These receivers are now integrated into billions of manned and unmanned commercial and military devices; however, GPS signals are susceptible to a wide variety of factors and may not always be available or trustworthy. Positioning accuracy can be significantly degraded in complex propagation environments that induce multipath fading or signal shadowing effects. These navigation signals can also be degraded or completely disrupted by intentional or unintentional interference. For example, GPS signals are vulnerable to intentional jamming and spoofing attacks, which can lead to a loss of positioning and navigation integrity. These issues can lead to significant vulnerabilities in commercial and military infrastructure and the degradation or malfunction of vehicles and systems, such as UASs.

The loss of GPS signal integrity is a serious issue for manned and unmanned systems; however, it is particularly problematic for unmanned systems. A human operator can use other means for navigation if GPS-based positioning is unavailable in a manned system, including the use of on-hand information such as maps, landmark recognition such as street signs, the use of other navigation devices such as a magnetic compass, or collaboration with others, such as asking others for their current location. Human operators also have the cognitive means to fuse these alternate data sources to provide a reliable position estimation. Unmanned systems may have access to additional data sources, other sensors, and perhaps even collaboration opportunities with other manned or unmanned systems; however, they must possess the intelligence to process and fuse this information to estimate position autonomously. Furthermore, unmanned systems come in various form factors and cost points, which may limit the types of onboard sensing or processing available for positioning and navigation.

Many studies reported in the literature have attempted to characterize the performance of GPS positioning in complex propagation environments and proposed methods to augment GPS-based positioning with other sensor types that may be onboard a system, such as visual methods using an onboard camera. Numerous approaches have been proposed to provide autonomous methods to detect or mitigate the effects of hostile jamming and spoofing. Furthermore, many non-GNSS methods of positioning and navigation in GPS-denied environments exist. Many surveys and systematic reviews on GPS security and alternate positioning and navigation methods have been published; however, many only briefly discuss GPS security within a larger cybersecurity discussion. Other reviews focus on niche topics related to GPS security or alternative non-GNSS positioning and navigation methods. There are no existing comprehensive reviews of GPS security issues in the literature. This paper fills that gap by providing a comprehensive treatment of GPS security.

We provide an overview of the factors that can degrade or disrupt GPS signals in this paper, particularly those related to cybersecurity. Section 2 summarizes previous surveys and systematic reviews related to GPS security. Section 3 introduces GPS and establishes the performance baselines of GPS systems in benign conditions. Section 4 discusses the various disruption scenarios, attack types, and threat systems that can affect successful GPS operations. Section 5 provides an overview of GPS jamming and spoofing detection methods. Section 6 discusses countermeasures to GPS jamming and spoofing, including an analysis of the state-of-the-art in positioning and navigation in a GPS-disrupted environment. Finally, Section 7 provides conclusions and open research directions. This paper focuses on GPS positioning and navigation aspects and does not cover the timing aspects of GPS performance or alternative timing approaches in GPS-denied environments. Furthermore, this paper considers generalized GPS performance, focusing on positioning and navigation within UAS applications.

Many modern devices do not rely solely on GNSS systems such as GPS; they rely on multiple sensors working in conjunction with GNSS for positioning and navigation. These sensors can sense the system’s attributes, such as with inertial sensors, or the surrounding environment’s attributes, such as with cameras. Data from these sensor systems are fused with GNSS-based data using methods such as a Kalman filter. Other systems utilize multiple GNSS systems, including GPS, GLONASS (Russian GNSS), Galileo (European GNSS), and Beidou (Chinese GNSS). The loss of GPS does not represent a complete loss of navigation data in multi-sensor or multi-GNSS systems but rather represents the loss of a single data source that could cause performance degradation to positioning and navigation solutions.

This paper extensively discusses various sensors and associated algorithmic methods used for positioning and navigation instead of providing a comprehensive examination of all components within a complex multi-sensor or multi-GNSS navigation system; however, its primary focus lies in GPS-denied scenarios, exploring these sensors and methods as strategies to counter GPS vulnerabilities and ensure reliable positioning and navigation even under compromised GPS conditions.

## 2. Existing Surveys

Several surveys related to GPS performance, GPS security, and navigation and positioning in GPS-denied and GPS-disrupted environments can be found in the literature. Table 1 lists recent surveys related to positioning and navigation in GPS-denied environments, emphasizing UAS applications, and specifies the topics covered and not covered by each of these surveys.

These surveys are analyzed in terms of (1) type of attack, (2) attack detection method, and (3) attack mitigation approaches. The papers were categorized according to the final topics: (1) GPS spoofing, an attack type where a malicious actor attempts to broadcast a falsified GPS signal to trick a receiver into calculating an incorrect position; (2) GPS jamming, an attack type where a malicious actor attempts to broadcast an interference signal to prevent a receiver from properly receiving the actual GPS signal; or (3) alternative (non-GNSS) positioning and navigation, such as positioning methods in GPS-denied environments. The authors of [1,2,3,4] provided a holistic review of literature focused on the UAS cybersecurity space, encompassing general aspects related to UAS cybersecurity, including GPS jamming and spoofing. They also analyzed the overall cybersecurity challenges facing unmanned systems, covering a broad range of threats to the various sub-systems that comprise these systems. The authors of [5] provided a comprehensive overview of jamming attacks and mitigation strategies across a wide range of wireless technologies, including GPS receivers. The authors of [6] focused on GPS jamming, summarizing different types of attacks and mitigation approaches. The authors of [7] presented a comprehensive review of unintentional and intentional threats against GNSS systems, focusing on GPS, discussing various degradation mechanisms, attacks against the GPS system, and many types of mitigations. The authors of [7] assessed the impacts of various types of degradation in terms of accuracy, integrity, availability, and continuity. Mitigation strategies were analyzed in terms of performance, cost, and complexity. The authors of [8,9,10] presented comprehensive reviews of GNSS spoofing threats, focusing on GPS. There is overlap between these surveys in the approaches they summarize, but they use different attack and mitigation taxonomies, which reflect slightly different foci between these papers. The authors of [8] proposed a taxonomy of spoofing mitigation methods consisting of (1) signal processing-based methods, (2) encryption-based methods, (3) correlation-based methods, and (4) antenna-based methods. The authors of [9] used a different spoofing mitigation taxonomy, dividing approaches into (1) signal processing-based approaches, (2) encryption-based approaches, (3) drift monitoring-based approaches, (4) signal geometry-based approaches, and (5) multi-pronged spoofing defense approaches. The authors of [10], primarily considering UAS applications, focus primarily on different types of GPS attacks. However, the authors of [10] provide a summary of many methods to make receivers robust against spoofing attacks, including a variety of approaches based on GPS signal characteristics and the perceived source location of the GPS signal. The authors of [10] also discuss the potential benefits of multi-GNSS receivers to mitigate GPS spoofing attacks.

The other survey papers [11,12,13,14,15,16,17,18,19,20,21,22,23,24,25,26] described non-GPS methods of positioning and navigation. There are many surveys on navigation and positioning in GPS-denied and GPS-disrupted environments; however, these surveys typically focus on the narrow aspects of positioning and navigation based on specific sensor types or methods. Many surveys focused on aspects of Simultaneous Location and Mapping (SLAM), discussing how autonomous systems learn and map an area while determining position within that area. SLAM most commonly uses some combination of optical sensors, such as cameras and Light Detection and Ranging (LiDAR) sensors. Many papers in the literature focused on the visual methods of positioning, where a system uses a camera to detect known landmarks within its surrounding environment. Other surveys focused on Inertial Measurement Unit (IMU)-based positioning approaches, where sensors such as accelerometers and gyroscopes are used to track a system’s motion to determine relative position. Other papers in the literature focused on RF-based methods of positioning and satellite-based (non-GNSS) approaches. Several surveys related to positioning in GPS-denied environments focused more on summarizing the approaches found in the literature and did not provide a performance analysis of the approaches, discuss research gaps, or provide recommendations for future research directions. Table 2 provides a summary of those surveys that focused on positioning and navigation in GPS-denied environments and categorizes these papers in terms of the type of survey, creating a taxonomy with three high-level categories: (1) summarizing approaches in the literature on positioning and navigation in GPS-denied environments; (2) analyzing the effectiveness of alternative positioning and navigation methods; and (3) providing insight into necessary research directions based on previous studies. These papers are also categorized by each survey’s technical focus. 

Visual SLAM, or vSLAM, has received the most attention in existing reviews. The authors of [11,12] provided comprehensive reviews of GNSS-independent navigation methods for autonomous vehicles, and further reviews of vSLAM methods are prevalent in the literature. Many vSLAM-focused reviews significantly overlap the techniques they discussed and simply present them within different taxonomies; however, several surveys focused on different aspects of vSLAM. The authors of [11] focused on assessing the technology maturity and reliability of various SLAM methods, including visual and laser-based approaches, with IMU sensor assistance for two-dimensional and three-dimensional mapping applications. The authors of [12] reviewed existing methods within a taxonomy of map-based navigation, where a region’s map is known a priori and the system must determine its position within the known map, and mapless navigation, where little-to-know information about the region is known a priori. The authors of [13] presented one of the few reviews that discuss absolute visual localization (AVL) techniques, with a focus on UAS applications. AVL techniques provide absolute location in the form of latitude and longitude instead of relative location within some arbitrary reference system. Most existing surveys focused on relative visual localization (RVL) methods, in which the system’s location is only determined within a non-absolute reference system, typically relative to other objects or waypoints. The core issue surrounding RVL is error accumulation, also known as drift over time. This issue can be somewhat alleviated through IMU-based enhancements; however, these approaches do not correct the underlying problem or applications that require long-term precision positioning. The authors of [14] discussed approaches for specific functions within the overall vSLAM problem space, including obstacle detection, obstacle avoidance, and path planning, within the context of mapless navigation approaches such as performing navigation, without a known map, map-based navigation approaches, and map-building approaches. The authors of [15] focused on summarizing various path planning and obstacle avoidance algorithms from the literature, comparing the performance of various algorithms in terms of computational efficiency and the optimality of solutions for several scenarios and obstacle layouts. The analysis presented by the authors of [15] demonstrated that certain algorithms performed better in certain scenarios; however, there were cases when they had higher computational time or less optimal solutions than other techniques. The proper choice of algorithm should be based on operational requirements to best balance computational time and solution optimality. The authors of [16] provided a review of vSLAM and generalized vision-based navigation, focusing on visual methods for (1) map-based navigation systems, (2) obstacle detection and avoidance approaches, and (3) path planning-based approaches, highlighting the strengths and weaknesses of the various approaches. Future research challenges identified include (1) the need for improved scalability, (2) improved computational efficiency, (3) improved algorithm reliability, and (4) improved algorithmic robustness.

Several vSLAM reviews discussed methods within a taxonomy based on the type of onboard visual sensor. The authors of [17] focused on approaches using monocular cameras. The survey discussed traditional visual SLAM methods, such as LSD-SLAM, ORB-SLAM, MSCKF, and DL-based methods. The authors stressed the difficulties of achieving real-time capability in vSLAM approaches and pointed out several key research directions in visual SLAM, including (1) the need for combined approaches that utilize IMU-based techniques, (2) the need for more work in incorporating DL-based techniques, and (3) the necessary mitigation of feature dependence, which the authors argue is the greatest limitation of vSLAM. The authors of [18] created one of the most comprehensive vSLAM reviews found in the literature, summarizing vSLAM methods across different types and numbers of visual sensors, including monocular vSLAM, stereo vSLAM, and RGB-D vSLAM. The authors also discuss different vSLAM methods, including event-based vSLAM, multi-modal vSLAM, and visual–inertial SLAM. They categorize vSLAM systems into three types based on how they use information from images: (1) direct or dense methods, (2) feature-based methods, and (3) semantic scene understanding methods. Each of these methods is described in detail, and various approaches are presented and discussed. The authors of [19] presented a brief survey of vSLAM methods, focusing on monocular techniques and presenting a qualitative and quantitative performance comparison of three of the most prominent algorithms in the literature: ORB-SLAM2 (sparse feature-based method), LSD-SLAM (semi-dense direct method), and DSO (sparse direct method). The algorithms demonstrated good performance; however, monocular approaches are susceptible to lens flare, overexposed images, water reflections, and moving objects and should be supported by other sensors, such as an IMU.

Many reviews discussed individual vSLAM [18] or Lidar-SLAM [11] methods; however, they focused on multi-sensor fusion methods, where multiple orthogonal sensor outputs were fused to estimate position information. The authors of [22] reviewed vSLAM and LiDAR-SLAM approaches combined with IMU-based methods. The multi-sensor fusion techniques discussed include visual–inertial approaches, LiDAR-inertial approaches, visual-LiDAR approaches, and LiDAR-visual–inertial approaches. The authors identified several critical future research directions, including the need for a versatile and efficient sensor fusion framework, additional DL-aided methods, and distributed cooperative methods. The authors of [23] surveyed multi-sensor fusion methods combining vSLAM with IMU sensors and proposed a modular multi-sensor data fusion technique. The authors of [20] considered LiDAR SLAM and vSLAM, along with multi-sensor methods such as LiDAR-vSLAM, visual–inertial SLAM (VI-SLAM), and LiDAR-inertial SLAM. These SLAM methods were considered for different sensor types, such as monocular vs. stereo. The authors of [10] provided a comprehensive review of visual and LiDAR-based SLAM techniques and approaches, considering map and mapless approaches that perform tasks such as obstacle avoidance, path planning, and map generation. They discussed some methods that also incorporate IMU-based measurements together with visual and LiDAR methods. The authors of [24] reviewed LiDAR-based SLAM techniques, considering both 2D and 3D LiDAR-SLAM approaches, and described the technical challenges of 3D LiDAR-SLAM, specifically the typical low vertical resolution and sparse point clouds associated with LiDAR. They concluded that multi-sensor fusion methods are required. The general benefits of multi-sensor fusion approaches were discussed, focusing on inertial-aided LiDAR-SLAM and visual-aided LiDAR-SLAM.

Several reviews focused on using Artificial Intelligence (AI) and machine learning (ML) in vSLAM and Lidar-SLAM. The authors of [25] provided a brief survey of vSLAM with a focus on deep learning (DL)-based methods, pointing out four key advantages of DL-based approaches to vSLAM compared with traditional approaches, which they broadly categorized as feature point methods or direct methods. First, DL methods had good invariance to illumination changes compared with traditional approaches. Second, DL-based vSLAM approaches could better identify moving objects in images than traditional approaches. Third, high-level semantic information could be extracted through DL to provide better context and understanding in map creation. Last, DL-based approaches removed the need for hand-constructed feature generation, widely understood as a general strength of DL approaches in any application. The authors discussed many of the DL approaches that researchers have already incorporated into the overall visual SLAM system, including the VO, the closed-loop detection process, and the semantic SLAM module, highlighting the performance benefits of these methods in dynamic environments. The authors of [26] also focused on AI-based approaches for UAS navigation. The techniques highlighted in [26] employed various types of optimization-based approaches, including genetic algorithms (GA), particle swarm optimization (PSO), ant colony optimization (ACO), simulated annealing (SA), pigeon-inspired optimization (PIO), Cuckoo Search (CS) algorithms, Dijkstra’s Algorithm, Differential Evolution (DE), and Grey Wolf Optimization (GWO). The authors of [26] also discussed various DL-based approaches, including reinforcement learning (RL) and deep reinforcement learning (DRL). These algorithmic approaches can be applied to any navigation-related problem; however, the survey focused on path planning and optimization applications. The authors identified federated learning (FL) as a top future research direction, where AI model training occurs in a distributed manner across multiple devices using local datasets. Additional identified future research directions included the need for improved energy consumption, reduced computational power requirements, improved fault handling, and the need for AI-based solutions for physical threat avoidance.

The primary contribution of this paper is that we cover all aspects of Table 1 and Table 2. There are many high-quality surveys in the literature; however, they typically focus on niche areas of the problem space. This paper covers all relevant aspects of GPS security, including threats, attack detection, and attack mitigation for jamming and spoofing. This paper also comprehensively describes positioning and navigation in GNSS-denied environments.

## 3. GNSS Overview

### 3.1. GPS System

**Figure 1:**
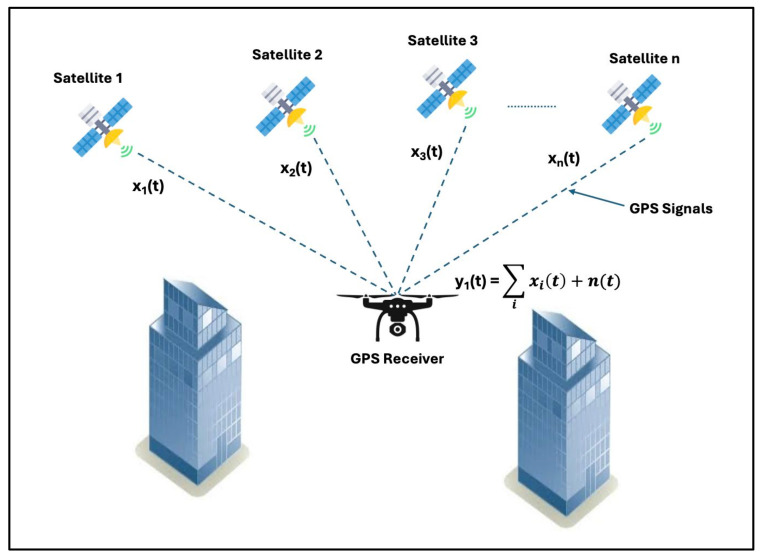
GPS trilateration for position estimation.

The GPS constellation comprises 31 satellites operated by the United States Air Force (USAF). The first GPS generation achieved full operational capability in 1995 and has been subsequently modernized with GPS Block III satellites, which began launching in 2018. GPS works through a process known as trilateration, where the location of one point in space can be determined by the known characteristics of at least three other points in space. Each GPS satellite transmits a unique signal that can be received by a GPS receiver on Earth’s surface. Embedded in that unique signal are the satellite’s current location and the absolute time that the satellite transmitted the message. The GPS receiver then estimates how long the message took to travel from the satellite to the receiver, providing the receiver with an estimate of the distance between itself and that satellite. A 2D position estimate can be calculated once the distance is estimated from at least three satellites. A 3D position estimate can be calculated with range estimations from at least four satellites through a process known as trilateration. Figure 1 depicts the GPS trilateration process. 

GPS satellites broadcast multiple downlink signals, including the L1 and L2 carriers operating at 1575.42 MHz and 1227.60 MHz, respectively. Newer GPS satellites also broadcast additional carriers, such as the L2C, L5, and L1C carriers. The L2C carrier was introduced in 2005 and gets its name from the frequency it uses (1227 MHz, the same as L2) and the fact that it is intended for civilian use. The L2C carrier is broadcast at a higher power than the L1 carrier, with the goal of better penetration through trees and buildings. The L2C carrier, sometimes referred to as the second civil signal, began broadcasting civil navigation (CNAV) messages in 2014; however, the USAF still considers L2C to be pre-operational. The L5 carrier, the third civil signal, is broadcast at 1176 MHz and was first introduced in 2010, with CNAV messages broadcast beginning in 2014. The L5 carrier is reserved exclusively for aviation safety services and features higher power and an advanced signal design for increased robustness compared with other carriers. L5 is still considered pre-operational by the USAF. The L1C carrier, referred to as the fourth civil signal, is broadcast at 1575 MHz and was originally designed as a common civil signal for GPS and Galileo. This carrier was designed to improve mobile reception in urban environments. Other GNSSs, such as BeiDou, are adopting similar signals. The first GPS satellites capable of broadcasting L1C were launched in 2018; however, L1 and L2, now known as the GPS legacy signals, remain the most common carriers supported by all PS satellite generations. There is also an L3 carrier at 1381.05 MHz, which is not used for navigation purposes. This carrier is used by the United States Nuclear Detonation (NUDET) Detection System (USNDS) to detect and locate nuclear detonations in the Earth’s atmosphere and is used primarily for enforcing nuclear test ban treaties. The GPS signal is a spread-spectrum signal that simultaneously transmits multiple types of ranging and navigation messages. The GPS employs the Binary Phase Shift Keying (BPSK) digital modulation scheme for transmission. Some GPS satellites also employ a form of Quadrature Amplitude Modulation (QAM). 

Each GPS satellite has several identifiers that are conveyed via ranging and navigation messages, including the space vehicle number (SVN), the space vehicle identifier (SVID), and the pseudorandom noise number (PRN). The PRN identifies which range code the satellite is using. A fixed unique mapping exists between the SVN, SVID, and PRNs described in the GPS interface specification [27]. The L1 and L2 carriers carry Course Acquisition (C/A) PRN codes, which are Gold codes transmitted at a rate of 1.023 Mbps and repeated every one millisecond. These C/A codes are exclusive or’d with a 50 bps navigation message containing information on the time and the satellite’s position. Each satellite’s unique PRN code is orthogonal to all other PRN codes, meaning each PRN code will not correlate with any other satellite’s PRN code. Each C/A code chip corresponds to 293 m of distance; therefore, the receiver tracking this code will result in a range estimation no worse than 293 m and better in most cases. The Precision Code, or P-code, is a sequence of 6.187104 × 10^12^ chips transmitting at a rate of 10.23 million chips per second (Mcps) and repeating once a week. Receivers can use the C/A code for course range estimation and the P-code for higher resolution range estimation. 

**Figure 2:**
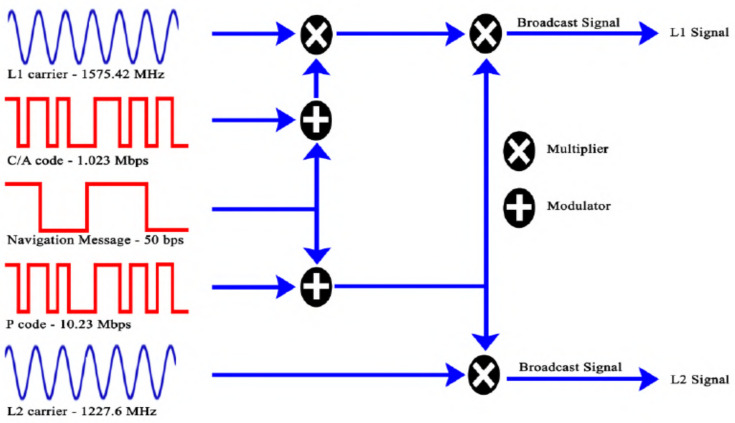
Legacy GPS signaling and messaging structure.

The original (legacy) GPS signal and messaging structure are depicted in Figure 2 [28]. The L2 carrier also has a W-code that is applied to the P-code at approximately 500 bps, the details of which are secret. This code is meant for US military usage; however, modern two-channel commercial GPS receivers can also track the L2 signal without knowing the W-code. These commercial receivers are more expensive and uncommon in consumer applications. A secure M-code in newer Block III GPS satellites is used for military applications that aim to improve the anti-jamming and secure access of military GPS signals.

Receivers will demodulate the BPSK signal and process the NAV message to determine the time and location of the transmitting satellite. The receiver can then estimate the transmission delay and consequently estimate the range of the satellite. The receiver can estimate its 3D position once it can receive at least four satellite transmissions and estimate the range of each satellite. 

GPS receivers utilize different aspects of the GPS signal to estimate range and position. Receivers utilize two primary observables to estimate the distance between the satellite and receiver: (1) code and (2) phase. The process previously described is code-observable; however, accessing the NAV message does not generally yield highly accurate results. This level of accuracy may be sufficient for some applications; however, additional precision can be obtained through the phase observable, where the unmodulated carrier phase is estimated by the receiver. Receivers can use the Doppler shift as another observable. The type and sophistication of processing vary across receivers based on several factors, including the required accuracy and desired cost point. 

The position estimate is presented as its solution, along with several metrics that convey confidence in that estimate once the receiver has processed the signals. The receiver provides the Dilution of Precision (DOP) metric and is intended to reflect the quality of the satellite geometry and the resulting data uncertainty. Position DOP (PDOP) is another key metric that reflects the uncertainty of the overall position estimate. Horizontal DOP (HDOP) and Vertical DOP (VDOP) reflect the uncertainty in the horizontal and vertical components of the 3D position estimation. Higher values of DOP and PDOP generally mean a more accurate position estimate. DOP and PDOP typically increase as the number of visible GPS satellites increases, such as when the number of GPS satellites for which the signal can be successfully demodulated and processed increases, the geometry of the GPS satellites is more advantageous, and GPS receive signal strength (RSS) is higher.

### 3.2. Other GNSS Systems

While GPS is the oldest and most mature GNSS system, there are other GNSS systems that have emerged since the GPS system was initially fielded. The first is the Russian Federation’s GLObalnaya Navigatsionnaya Sputnikovaya Sistema (GLONASS), with the first satellites launched in 1982 and reaching full operational capability in 1995. GLONASS consists of 24 satellites in medium-earth orbit (MEO). The constellation operates in three orbital planes, with eight evenly spaced satellites on each plane. For a GLONASS receiver to calculate an accurate position estimate, the receiver must be in range of at least four satellites. providing the system originally. GLONASS satellites broadcast two types of signals: (1) an open standard-precision signal and (2) an obfuscated high-precision signal available only to authorized users. The L1 and L2 carriers utilize Frequency Division Multiple Access (FDMA), where individual satellites transmit at slightly different frequencies. The most common GLONASS signals, L1 and L2, are centered at 1602 MHz and 1246 MHz, respectively, with both standard-precision and obfuscated high-precision signals provided at both L1 and L2. Starting with newer GLONASS-K1 satellites, a new L3 channel centered at 1207 MHz was introduced, which is also available on modern GLONASS-M+ satellites launched in the past decade. Similar to GPS, GLONAS uses Code Division Multiple Access (CDMA) and BPSK modulation. New CDMA-based signals have also been defined for L1 and L2 channels that are available in the newest GLONASS-K2 satellites.

Galileo, developed and maintained by the European Space Agency (ESA), is the European Union’s (EU’s) GNSS that consists of 28 satellites, all but two of which are positioned in three MEO orbital planes. The remaining two satellites were placed in incorrect orbits and are currently used for search and rescue purposes only but are not considered an operational part of the constellation. Galileo began providing an initial operational capability in late 2016. It broadcasts signals in three primary bands: E1, E6, and E5ab. The E1 carrier offers open service (OS) using a composite binary offset carrier (CBOC) modulation designed for reduced ranging noise and enhanced multipath performance. This E1 OS signal was also designed to provide improved interoperability with other GNSSs, sharing a common spectrum with the GPS L1C and BeiDou-3 B1C carriers. Dual-frequency receivers can also utilize the E5a and E5b signals, which share the same spectrum as the GPS L5 and BeiDou B2a carriers. The E5a and E5b carriers utilize an Alternative Binary Offset Carrier (AltBOC) modulation and multiplexing scheme. The E6 carrier includes a fully encrypted signal component for authorized users.

The Chinese BeiDou-3 system provides global GNSS service through its 24-satellite constellation in MEO across three orbital planes. Precedessor BeiDou systems (e.g., BeiDou-2) were regional systems and did not provide global service. BeiDou-3 began offering initial services in late 2018, with a full operational capability announced in 2020. This BeiDou-3 system offers four primary signals for navigation. The B1I and B3I carriers provide open services and were retained from BeiDou-2 for backwards compatibility. It also introduced the B1C and B2A carriers. B1C operates in the same spectrum as the GPS L1 and Galileo E1 carriers (1575.42 MHz), and the B2A carrier operates in the same spectrum as the GPS L5 and Galileo E5a carriers (1176.45 MHz). These spectrum choices were made to facilitate multi-constellation receivers. The BeiDou-3 system uses a range of modulation and multiple access approaches that are quite different than GPS, Galileo, or GLONASS systems. BeiDou-3 uses Constant Envelope Modulation via Intermodulation Construction (CEMIC) in the B1 band to generate the legacy B1I carrier. The B2A carrier is generated through Asymmetric Constant Envelope Binary Offset Carrier (ACE-BOC) modulation, while the B1C carrier uses Quadrature Multiplexed BOC (QMBOC). A unique feature of BeiDou-3 is the introduction of inter-satellite crosslinks that are capable of providing ranging measurements both within and across satellite orbital planes. This capability aims to reduce orbital errors, mitigate stale ephemeris, and consequently provide enhanced accuracy.

**Figure 3:**
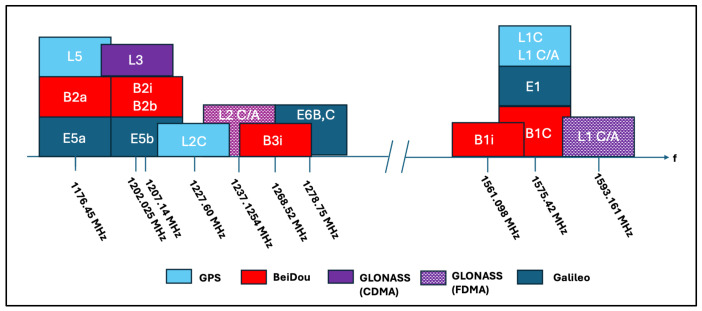
Spectrum allocations for worldwide GNSS civilian navigation signals.

While there are many key differences between these various systems, the operating principles are similar. They are all predicated on a receiver estimating the range between itself and each observable satellite and using that information to generate its self-position estimate. Each system has code observables, phase observables, and Doppler frequency observables. For more discussion about the subtle differences between these systems in terms of design choices and services offered, the authors of [29,30] provide an overview and comparison between several of these systems. Note that the authors of [29] limit their comparison between GPS and GLONASS, while the authors of [30] consider all four of these systems. Figure 3 shows the spectrum of these various systems relative to one another [30].

### 3.3. GNSS Performance Expectations

GNSS positioning accuracy can vary based on receiver complexity, such as the type of signal processing; variation in receiver quality, such as hardware quality; environmental conditions; location; time of day; and geometry. GNSS receivers actually do not determine a range to each satellite but rather a pseudorange, which is just an estimate of range. Ideally, this pseudorange, $\tilde{\rho }$, would match the actual range to the satellite, *ρ*, and would be a function of the speed of light, *c*, the time, *t_R_*, and the time of the transmitter clock, *t_T_*, such that $\tilde{\rho }=c\left(t_{R}-t_{T}\right)=\rho (t_{R},t_{T})$ [31]. However, in practice, this range estimation varies from the actual range due to a variety of degradation factors, including atmospheric effects, multipath propagation effects, clock errors, Earth tides, and relativistic effects, among other noise terms. Because of these various degradation factors, the pseudorange observable takes the following form [31]:

(1) $$\tilde{\rho }=\rho \left(t_{R},t_{T}\right)-c\left({\delta t}_{R}-{\delta t}_{T}\right)+\delta_{IONO}+\delta_{TROPO}+\delta_{TIDE}+\delta_{PATH}+\delta_{REL}+\varepsilon$$

where

δt represents clock errors;
$\delta_{IONO}\;$ and $\delta_{TROPO}\;$ represents atmospheric effects of the ionosophere and troposphere;
$\delta_{TIDE}$ represents errors introduced by Earth’s tidal cycles;
$\delta_{PATH}$ represents errors introduced by multipath propagation;
$\delta_{REL}$ represents relativistic errors;
ε represents all other unmodeled error sources [31].

Clock errors can be present on the satellite or receiver and can be caused by ephermis errors, receiver clock drift and bias, and measurement error. Ionospheric delay is a function of electron density along the signal’s propagation path. Tropospheric delay is a function of environmental conditions along the signal’s propagation path, such as temperature, barometric pressure, and humidity. Trophospheric propagation also introduces signal attenuation based on conditions. Multipath fading introduces delays due to signals traveling different propagation paths and can also lead to large-scale and/or small-scale fading (i.e., constructive and/or destructive combining at the receiver of signals taking different propagation paths). Other sources of error can include factors such as receiver noise, external noise/interference, and other propagation effects (e.g., blockage) that reduce the quality of the received signal.

As previously mentioned, some GNSS receivers make carrier-phase measurements of the GNSS signal to gain higher precision than is available through the code observable. However, there are error components that arise in this measurement. Two issues surrounding phase measurement are (1) phase ambiguity and (2) cycle slip. Phase ambiguity is due to the fact that adding integer multiples of the signal cycle will result in exactly the same measured phase. There are numerous methods that deal with phase ambiguity in GNSS receivers. But generally, these receivers do not distinguish between carrier cycles and generally measure fractional phases and then track phase changes. GNSS receivers will attempt to estimate an unknown initial ambiguity from the GNSS data. However, errors are generally present in the phase measurement. Cycle slip refers to the need for GNSS receivers to continually track signal phase; intermittent outages (e.g., signal shadowing) will change the value of the phase ambiguity. In this case, carrier phase tracking must start over, and there will be a period of time with degraded performance.

If there are no errors and no propagation effects, the measured phase, Φ, will take the following form [31]:

(2) $$\Phi =\Phi_{R}\left(t_{R}\right)-\Phi_{T}\left(t_{R}\right)+N_{R}^{T}$$

where

$\Phi_{R}$ is the phase of the receiver;
$\Phi_{T}$ is the phase of the received satellite signal;
$N_{R}^{T}$ is the ambuiguity between the satellite and receiver.

However, as was the case in the previous pseudorange discussion, the measured phase will differ due to several degradation factors, and the carrier phase measurement model will take the form of Equation (3) [31].

(3) $$\lambda \Phi =\rho \left(t_{R},t_{T}\right)-c\left({\delta t}_{R}-{\delta t}_{T}\right)+\lambda N_{R}^{T}-\delta_{IONO}+\delta_{TROPO}+\delta_{TIDE}+\delta_{PATH}+\delta_{REL}+\varepsilon$$

where λ is the signal wavelength, and the remainder of the terms are similar to those in Equation (1). For a more thorough description of the various pseudorange and carrier phase measurement models and error components, the reader is referred to [32].

#### GPS Performance

The formal GPS performance standard published by the United States Department of Defense (DoD) [33] states that “[…] well-designed GPS receivers have been achieving horizontal accuracy of 3 m or better and vertical accuracy of 5 m or better 95% of the time”. This level of accuracy has been substantiated by numerous studies, several of which are summarized in Table 3; however, these studies established variation due to the factors mentioned above.

Multi-frequency GPS receivers processing both L1 and L2 carriers can obtain improved position accuracy with centimeter-level accuracy [41]; however, as previously noted, multi-frequency GPS receivers are larger, more expensive, not common in consumer or commercial applications, and are historically more prevalent in professional and military applications.

There is also the concept of Differential GPS (D-GPS). D-GPS is based on the idea that two receivers near each other experience similar atmospheric errors. A network of GPS receivers at known locations is established, and they publish their signal measurement data for each visible satellite for public consumption. GPS receivers can then download this information, commonly referred to as GPS correction data, from the nearest fixed site and apply that data to correct any measurement errors in their data, improving the accuracy of their position estimation. This process can be done in real time if the GPS receiver can access the required connectivity. More commonly, differential correction is performed in non-real time by post-processing the published correction data.

## 4. Factors Contributing to GPS-Denied or GPS-Disrupted Environments

Several factors can contribute to GPS degradation or disruption, either intentionally through malicious attacks or unintentionally from unintended interference or propagation-related degradation. These factors include multipath fading, signal shadowing, unintentional interference, jamming, and spoofing. These various degradation factors have different degrees of implementation difficulty, require different levels of hardware and software complexity, and require varying levels of technical expertise to implement, making different attack scenarios more likely. Additionally, these various degradation factors have key differences in terms of potential effect, scope of the potential effect, and different potential ramifications. The various types of GPS degradation factors and their key characteristics are summarized in Table 4. 

### 4.1. Propagation-Induced GPS Degradation

**Figure 4:**
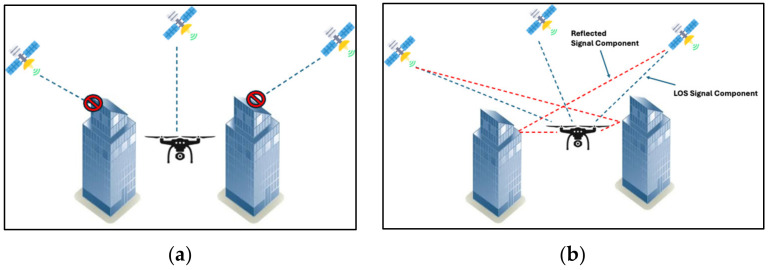
Propagation-induced GPS degradation factors, including (**a**) signal shadowing and (**b**) multipath fading.

The very low GPS power levels received at the Earth’s surface make the system sensitive to blockages and shadowing. This issue occurs when a manmade structure or natural terrain feature blocks the line-of-sight (LOS) from one or more GPS satellites to the receiver. The need for the precise propagation time estimation required to estimate receiver-to-satellite range for GPS trilateration also makes the performance sensitive to multipath fading propagation conditions. This problem occurs when the GPS signals reflect off manmade structures toward the GPS receiver. The reflected signal components will be delayed in time compared with the LOS signal component due to the longer propagation path taken, resulting in the GPS receiver receiving the LOS component of the GPS signal and these time-delayed reflected signals. These different time-delayed versions of the same signal can either constructively or destructively add at the receiver, depending on the geometry and environment, resulting in large-scale and small-scale signal fading. These degradation factors are problematic in both suburban and (particularly) urban settings. The “urban canyon” scenario, which describes the scenario within a large urban area with many tall buildings, has historically proved problematic for GPS signal reception. These propagation-induced degradation factors are depicted in Figure 4.

Several studies have investigated GPS performance in suburban and urban propagation environments; however, these studies do not typically isolate degradation due to shadowing or multipath fading. Instead, both shadowing and multipath fading are loosely categorized as urban propagation. GPS performance in complex urban environments has been the focus of study in several existing papers [35,37,42,43] with greatly varying reported results. The authors of [43] presented experimental results revealing position accuracies of less than five meters in a built-up urban environment, similar to those observed in the literature for open environments. The authors of [42] also observed good performance in an urban measurement campaign, with reported accuracies of 7–13 m; however, the authors of [37] observed much greater variability in position accuracy in urban environments. The authors of [37] concluded that location-based applications must assume a position error of at least 28 m within their application due to the effects of signal shadowing caused by nearby buildings. 

The varying results reported in these research papers suggest that performance in urban environments is sensitive to environmental details and subject to high variability. These results could suggest that the precise effects of multipath fading and shadowing on GPS reception may not be easily generalized and might require study for specific regions of interest; however, other frequency bands and signals of interest, such as commercial cellular communications, have generalized channel models that are reasonable across a wide range of complex urban environments. These frequency bands have historically received significantly more interest, with thousands of papers published on cellular channel models and measurement campaigns; therefore, the lack of a high-fidelity generalized GPS urban propagation model may be due to insufficient research and publications. Researchers were in general agreement that urban canyon scenarios, such as very tall buildings in dense city downtowns, yield poor accuracy performance or total GPS signal loss.

One problem with all studies on this subject is the lack of experimental details and subsequent lack of reproducibility. The papers on GPS performance in urban environments do not give a sufficient description of the details of their experiments or their test environments. It is challenging to draw generalized conclusions or validate the results of these papers without these details. Furthermore, it is difficult to conclude what factors provide the largest contribution to the results without an attempt to isolate degradation factors, such as shadowing and multipath fading.

### 4.2. GPS Jamming and Unintentional Interference

GPS signals are extremely weak on Earth’s surface, often on the order of −130 dBm or lower. A relatively low power emitter can interfere, intentionally or unintentionally, with proper GPS signal reception, exacerbated by the fact that commercial GPS receivers use varying-quality hardware components and signal processing methods, which can lead to poor performance for some receivers in non-pristine electromagnetic environments. Unintentional interference can be caused by malfunctioning or misconfigured equipment that emits energy into the GPS frequency bands. Unintentional interference can be caused by unsuppressed harmonic emissions into the GPS band or intermittent spurious out-of-band emissions from RF equipment. Unintentional interference can be difficult to detect or locate due to the highly temporal nature of the interference, especially if it is caused by mobile equipment or human activity. Intentional GPS interference, also known as GPS jamming, is typically one of four types: (1) barrage jamming, (2) continuous wave (CW) or tone jamming, (3) chirp jamming, and (4) protocol-aware jamming. Barrage jamming involves the transmission of noise across a wide bandwidth, covering the entire target signal’s channel bandwidth. CW jamming focuses the interference energy into a very narrow bandwidth within the target signal’s channel. Chirp jamming is an approach where the interference frequency changes rapidly over time. Chirp jammers are often narrowband; therefore, they resemble a swept-tone signal. Protocol-aware jammers attempt to utilize the target signal’s attributes to optimize their jammer waveform. 

There is wide agreement that GPS is susceptible to interference; however, only limited studies on the topic of GPS interference exist. The authors of [44] presented the results of a measurement campaign conducted across Europe over two months in 2016. The authors established eleven signal detection sites across seven European countries: the United Kingdom, Sweden, France, the Czech Republic, Poland, Slovakia, and Finland. Site locations included airports, near major roads, above motorways, busy city areas, and urban areas. Continuous spectrum monitoring and capture were conducted, and the resulting data were processed to determine interference events and attempt to classify the interference type. Over 5000 interference events were recorded over this period, with over 1000 classified as significant interference events, with positioning errors of hundreds of meters or complete GPS disruption near the receivers. The authors of [44] analyzed and identified the different types of interference events. Table 5 [44] summarizes the mean weekly occurrence rate for different types of measured interference events.

These various interference events had different durations and power levels. Many of these events were ultimately deemed unintentional interference, such as the wideband noise and narrowband signals in the two leftmost columns of Table 5. The detected chirp signals are likely from GPS jammers since chip signals are common for GPS jammers sold on the Internet (black market) [45,46]. The results of this type of measurement campaign vary across location and time; eleven randomly chosen locations had different results. Two important conclusions can be drawn from the results of this measurement campaign: (1) unintentional GPS interference is a common occurrence, and (2) intentional GPS interference, such as GPS jamming, is not a rare event.

A similar measurement campaign was performed by the authors of [47], primarily focusing on two locations in the Czech Republic over 140 days in 2021. During this time, 2158 interference events were reported, 872 of which were deemed high-impact events that caused GPS accuracy degradation or outages. One event was particularly interesting because it occurred simultaneously across both locations, which also strongly correlated to an interference signal recorded at test sites throughout Europe, including Belgium, France, Germany, Latvia, Finland, and the United Kingdom. This event was a narrowband interference signal located near the center of the L1 carrier and caused widespread GPS signal disruption or outage for several seconds. The estimated affected area was approximately one million square kilometers. Data were analyzed to determine if this event could have been caused by solar activity or some type of space weather event; however, the source of this interference remains unknown. 

Several papers in the literature discuss the feasibility of implementing low-cost GPS jammer systems. For example, the authors of [48] presented a study examining the detection and jamming of small commercial UAVs through low-cost commercial GPS jammers. The authors of [45] described results from a study that implemented a GPS attack system against a DJI Phantom 3 quadcopter UAS utilizing low-cost, commercially available hardware and software. The authors used a BladeRF X40 software-defined radio (SDR) platform with signal generation performed within the GNU Radio SDR development environment. The authors of [49] utilized a similar jammer design as reported in [50], using a BladeRF x40 SDR platform and the GNU radio software environment. They implemented various jamming techniques, including barrage jamming, CW tone jamming, sweeping noise jamming, sweeping narrowband pulse jamming, and a protocol-aware jamming waveform that matched the characteristics of the GPS signal structure. The authors of [51] demonstrated an SDR-based GPS jammer developed within GNU Radio. The authors of [52] also demonstrated an SDR-based GPS jammer developed using a BladeRF X40 SDR and GNU Radio. The results of [48] through [49] revealed that effective GPS jammers can be constructed using low-cost commercial (typically black market) GPS jammers or low-cost SDR hardware and software components. Effective GPS jamming systems are not difficult to implement using low-cost and no-cost open-source components. Table 6 summarizes the various GPS jammer implementations found in the literature.

A small number of papers quantitatively study the impact of jamming on GPS receiver performance. Most notable are the research efforts presented by the authors of [53,54]. The authors of [53] analyzed several different types of jamming against a simulated GPS waveform. They considered four different types of jamming strategies: (1) pulse jamming, (2) continuous wave (CW) jamming, (3) barrage noise jamming, and (4) swept partial-band noise (PBN) jamming. This study demonstrated that CW jamming results in the largest degradation of the GPS signal, closely followed by barrage noise jamming. BER performance asymptotically approached the BER rates as jammer power increased, likely due to the spread spectrum nature of the GPS signal. Further degradation was likely due to the saturation of the GPS receiver’s RF front-end hardware. The authors did not consider chirp jamming, which is known to be highly effective against GPS. Table 7 summarizes the effectiveness of the different jammer strategies presented in this study [53].

The studies presented in [54,55] provided insight into the performance variability of different GPS receivers in the presence of jamming. The authors of [54] presented an analysis of GPS performance against chirp jamming, commonly found in commercially available GPS jammers. The authors primarily considered linear chirp jamming against two GPS receivers and measured the corresponding GPS carrier-to-noise (C/No), DOP, and position solution accuracy. One of the test receivers was a multi-GNSS receiver; therefore, it was tested in GPS-only and multi-GNSS configurations. This study reported significant differences in performance between these GPS receivers, with some scenarios resulting in minimal error in one device and errors of up to 10 m in the other device. The authors also noted that the multi-GNSS configuration resulted in the best performance, with little-to-no performance degradation in the presence of GPS jamming. This result is expected since other GNSS systems, such as GLONASS, operate in different frequency bands, and the receiver always receives unaffected signals from at least one GNSS system. The authors of [55] conducted an empirical study of five different GPS receivers in the presence of jamming, attempting to gain insight into the variability of GPS receiver performance. This study presented the minimum jammer power levels required to make the different receivers lose GPS signal lock; however, the authors failed to describe the propagation path between the jammer and target receivers, and it is not possible to determine meaningful receiver metrics such as Signal Power to Jammer Power (S/J). 

The data from [55] provided insight into the variability of GPS receivers in jamming conditions. The five GPS receivers that were tested required jammer power ranging from −45 dBm to −60 dBm to disrupt their GPS signal reception. These results suggest a 15 dB variability in S/J performance across the five tested GPS receivers, assuming receivers were collocated such that GPS signal power was comparable across all receivers. This variability is significant and suggests that different GPS receivers will respond significantly differently to a jamming signal or unintentional interference. 

### 4.3. GPS Spoofing

Commercial GPS signals are not authenticated or protected from malicious attacks, including GPS spoofing, where an attacker broadcasts falsified GPS signals, allowing a receiver to believe they are in a different position. This type of attack is easy to generate due to the low signal strength of the downlink GPS signal, making it easier for the malicious attacker’s signal to overwhelm the actual GPS signal at the receiver. This attack type is more relevant to the L1 carrier since it does not have any type of protection and is less relevant to the L2 carrier since the L2 carrier implements cryptographically protected codes. Consequently, this type of attack is more relevant to commercial and consumer GPS receivers.

There are a limited number of papers that discuss real-world examples of GPS spoofing attacks; however, the authors of [56] did provide a discussion of some real-world examples, such as the well-known “Iran-US RQ-170 incident”, where Iranian forces captured a Lockheed Martin RQ-170 Sentinel UAS using a GPS spoofer in December 2011, which was publicly confirmed by the US military shortly after the event took place. The authors of [56] also discussed the work conducted by researchers from the University of Texas at Austin in 2013 and 2014. The authors built a GPS spoofer using low- and no-cost hardware and software components and then successfully spoofed a UAS and a ship. The spoofer caused the ship to travel in a zigzag motion while the ship’s autopilot reported a straight line of travel. The authors of [57] described the results of experiments conducted to seize control of a UAS navigation system through GPS spoofing. They systematically analyzed how to transmit falsified GPS signals and then prevent detection to maintain UAS control. The authors of [58] presented research where they implemented a GPS spoofer and injected the spoofed signal into a GPS receiver embedded within a UAS testbed. That GPS receiver was not providing stand-alone navigation but was rather part of a fusion approach that combined GPS data with inertial sensors. The authors demonstrated that even in this multi-sensor fusion approach, GPS spoofing was effective and that the UAS testbed’s navigation system was compromised. The study presented in [58] did not utilize an actual UAS but rather a testbed running the actual flight control software of a commercial UAS. So while the results must be caveated as such, these results still clearly illustrate the effectiveness of GPS spoofing.

GPS spoofers can be coherent, meaning they are phase and frequency synchronized with the actual GPS signal, or non-coherent, meaning they are not phase and frequency synchronized with the actual GPS signal. The authors of [59] presented a theoretical relationship between GPS spoofing signal synchronization error and required GPS spoofing signal power. The authors of [59] then provided practical advice and guidance on optimizing system design to GPS spoofing system designers. The authors of [60] also provided an analysis of potential GPS spoofer system design choices, focusing on potential techniques and strategies a spoofing system may employ in an attempt to minimize detection. Coherent GPS spoofing is more difficult to implement but can have a greater impact on the target receiver and requires stronger detection mechanisms within the receiver. The authors of [61] developed an advanced digital signal processor (DSP)-based GPS spoofer that was capable of high-fidelity civil GPS signal creation with significant carrier phase and Doppler frequency offset accuracy, such that the spoofed signal was virtually indistinguishable from authentic GPS signals. This work aimed to study the effectiveness of potential defense mechanisms against spoofing attacks. Many potential defenses were postulated; however, the authors concluded that cryptographic authentication was required against a sophisticated spoofing attack.

Many studies have established that low-cost GPS spoofers can be implemented using commercial SDR hardware and open-source software. A wide variety of low-cost SDR units are available, such as the HackRF and BladeRF platforms, which are the most popular. There are also open-source signal generation environments that can be leveraged for GPS spoofing, such as the GPS-SDR-SIM GPS signal simulation software package. The authors of [62] presented research in which they constructed a low-cost GPS spoofing system using a HackRF One SDR platform connected to a laptop computer running the GPS-SDR-SIM GPS signal simulator. This GPS spoofing system was tested against a Holystone HS7000 UAS. The authors did not provide results in terms of effective range, but they did demonstrate that spoofed signals are effective. The authors of [50] also used their BladeRF X40 SDR-based system to implement a GPS spoofing attack. The authors of [63] constructed a GPS spoofer utilizing the BladeRF x40 SDR platform with the GPS-SDR-SIM GPS signal simulator. They tested this low-cost GPS spoofer against a Huawei tablet with a GPS receiver and demonstrated that the receiver reported the spoofed GPS position. The authors of [64] presented a low-cost GPS spoofing system based on the HackRF One SDR platform attached to a laptop computer running the GPS-SDR-SIM GPS signal simulator. They tested the effectiveness of this solution against a GPS-enabled smartphone, successfully demonstrating that their low-cost system transmitting a spoofed L1 GPS carrier caused the smartphone to report the incorrect spoofed position. The research presented in these papers clearly illustrates that an effective GPS spoofing system can be implemented easily and inexpensively. Table 8 summarizes low-cost GPS spoofer implementations found in the literature.

There are several additional critical insights from existing research. First, unmanned systems can respond erratically and catastrophically to rapidly varying spoofed positions. The authors of [62] noted that a UAS exhibited erratic behavior, rapid speed increases, and a subsequent crash when a very low position altitude was rapidly spoofed. The authors of [65] discussed how rapid or erratic changes in the spoofed position could result in UAS crashes and proposed a GPS spoofing algorithm that implemented slow changes in the spoofed location to create the desired deception trajectory without adverse effects, such as crashing or detection. Second, a GPS spoofer’s effectiveness depends on the quality of the authentic GPS signal. Experiments performed in [50] examined attacks against a GPS-enabled smartphone device in indoor and outdoor scenarios. The GPS spoofing system successfully tricked the receiver in both scenarios; however, spoofing took significantly longer outdoors.

In some cases, GPS spoofing did not become effective until approximately three minutes later. Spoofing was never effective when the actual GPS signal had a very high DOP. These results suggest that the effectiveness of GPS spoofing might be directly related to the signal’s quality in terms of signal strength and system geometry. These results also suggest a temporal aspect to spoofer effectiveness. A spoofed GPS signal does not necessarily have to “overpower” the actual GPS signal to be effective. A key result from [63] is that position estimation error begins to grow if the spoofed GPS signal is 8.04 dB weaker than the actual GPS signal. The spoofed signal controlled the position once it was 4.52 dB weaker than the actual GPS signal. There is an example in the literature revealing the ease with which GPS timing information can be spoofed, such as when the GPS receiver has an accurate position but the attacker controls the absolute time information [66].

## 5. Detection Techniques and Their Comparison

The necessary first step in effectively mitigating performance degradation is reliably detecting that degradation mechanism. This section provides an overview of the detection mechanisms found in the literature for different types of GPS disruptions. We did not focus on propagation-induced GPS disruption; instead, we focused on detecting intentional attacks on GPS receivers, GPS jamming, and GPS spoofing. Some of the existing research efforts can apply to multipath-induced GPS disruption and unintentional interference scenarios.

### 5.1. GPS Jamming Detection

Most commercial GPS receivers do not have anti-jam or jamming detection features; however, there are some examples of commercial products that have jamming detection features. The authors of [55] presented a study evaluating the performance of various GPS receivers in the presence of jamming. One of the devices, the u-blox NEO-6 GPS receiver, was equipped with jamming detection capability. The NEO-6 provides a jamming level estimator (the jamIND field in the MON-HW message) that is intended to assess the likelihood of an ongoing jamming attack. A higher value indicates that a jamming signal is likely present. A narrowband jammer signal near the L1 center frequency interfered with the receiver and disrupted navigation; however, the NEO-6 reported an extremely low probability of a jamming attack. This result is unsurprising given the low-cost nature of most commercial GPS receivers. Existing commercial jamming detection capabilities are unlikely to perform well.

There is significant research on GPS jamming attack mitigation; however, there are far fewer papers on GPS jamming detection. Historically, simple energy detection and thresholding have been used for jammer detection. This approach works well for high-power jammer systems; however, energy-based approaches do not perform as well when the incident jammer energy is comparable to the target signal energy. Some of the papers that proposed GPS jamming mitigation approaches also embedded a form of jamming detection as part of their overall approach, but generally, those approaches are not stand-alone in nature. Overall, existing GPS jamming detection methods appear to fall into one of three primary categories: (1) based on the statistical properties of the received signal, a derivative of energy-detection approaches; (2) based on antenna array hardware approaches; and (3) based on ML approaches. 

Research has historically focused on the first two categories: statistical signal properties-based and antenna-based approaches. Newer proposed approaches have attempted to leverage ML-based approaches that can consider the GPS signal properties, the jammer signal, and the GPS receiver to support detection decisions. Open questions for these approaches are (1) how they perform in complex propagation environments, such as urban canyons, and (2) how they perform with GPS receiver equipment, given the variation in GPS receiver performance that has been demonstrated previously. Another consideration is the complexity of the proposed solutions and whether they are practical for small UAS platforms with limited computational capability. This question is particularly relevant for ML-based approaches and whether they can be computationally optimized to execute on unmanned platforms with low processing capabilities, small amounts of volatile memory, and limited persistent storage space. These approaches are summarized in Table 9. 

#### 5.1.1. Signal Statistics-Based Methods for Jamming Detection

Examples of these methods include using changes in signal strength, power spectral density, or other statistical properties to identify the presence of a jammer. The authors of [67] proposed a method to detect and classify the type of jamming signals based exclusively on the statistical properties of the power spectral density (PSD) for the overall received signal. The authors of [67] demonstrated that the PSD of the composite GPS plus jammer signal will take on different shapes and statistical properties. Specifically, the proposed approach attempted to characterize the range of maximum PSD amplitude values for the received signal and then used PSD amplitude to characterize jammer presence and type. The advantage of this approach is its simplicity and ease of implementation in real-world GPS receivers; however, certain jammer signal types, specifically swept noise jamming, have PSD characteristics like the GPS signal itself, which would appear to make it difficult to detect this jammer type. The method proposed in [67] also does not seem to account for propagation loss between the jammer and target GPS receiver. It is unclear how one would necessarily know the appropriate range of maximum amplitude values for the received jammer signal PSD without this knowledge. A similar approach was proposed in [68], where the statistical properties of the received signal spectrum were used to detect the presence of a jammer. The fundamental assumption in this approach was that the jammer caused any variations in the received signal’s statistics. Using this assumption, substantial changes in PSD mean or variance would be attributed to the presence of a jammer. It is unclear how this approach will perform in complex multipath fading and signal shadowing environments, where significant receive signal fluctuations will occur with or without the presence of jamming. Additionally, these approaches may perform well for high-power jammers, but it is unclear how they will perform when the received jammer power is roughly equivalent to the GPS signal.

The authors of [69] proposed a somewhat similar approach to detect the presence of a jamming signal based on the overall RSS in the L1 GPS band. BER was considered in the jammer detection approach. The underlying assumption was that a rapid change in RSS or BER was likely due to the presence of a jammer signal. The proposed approach monitored and created a time-series history of RSS and BER for the L1 band. Jammer detection was declared when a rapid increase in RSS and BER was detected. The results presented in [69] suggested very good detection performance for this approach; however, this approach may be limited in scope and may not perform well in multipath and shadowing propagation environments where the actual GPS signal level may be rapidly fluctuating. The research in [69] focused on the use case of an oceanic surface ship attempting to detect GPS jamming. The receiver would have clear visibility into the GPS satellite constellation and would not rapidly fluctuate in that environment. Consequently, it may be a reasonable assumption that any rapid increase in BER is caused by interference; however, this assumption may not hold true in a complex environment that produces multipath fading and signal shadowing. Rapid rises in BER could just as easily be caused by signal shadowing or destructive signal fading. Rapid rises in RSS could be the result of constructive signal fading. 

A similar approach was proposed by the authors of [70], where a Moving Variance (MV) approach was proposed to detect the occurrence of jamming in the L1 band. This approach was predicated on the assumption that rapid variations in L1 carrier-to-noise (C/No) Density Power are likely attributable to a jammer. A rapid change in the sliding window statistics of the L1 C/No was used to declare the presence of a jammer. As in the case with [69], it is unclear how this approach will perform in dynamic and complex multipath and signal shadowing environments, where C/No will be rapidly fluctuating regardless of the presence of a jammer signal. 

#### 5.1.2. Antenna Array-Based Methods for Jamming Detection

These approaches generally rely on using some statistical property of the received signal combined with spatial processing to detect the presence of interference. The authors of [71] proposed an antenna array approach based on measuring the carrier phase differences of incoming signals. Specifically, the double difference of the carrier phases was used to detect the presence of a jammer. The double difference in the carrier phases of these two signals will be extremely small if a jammer generates two PN code signals because they were generated from the same platform and arrived at the receive antenna from the same direction. Two PN code signals generated by actual GPS satellites will arrive from different directions and have a much larger double difference. This procedure can be simply implemented by measuring the carrier phase of the incoming signal and comparing it with a value that would be representative of a jammer-generated signal. The authors of [71] reported good detection performance; however, this type of approach would require an antenna array, which is more complex than the typical patch antennas found in most commercial GPS receivers. It is unclear if this approach would be feasible for smaller, unmanned platforms.

#### 5.1.3. ML-Based Methods for Jamming Detection

These approaches employ multiple properties of the received signal as features in a supervised ML model. The authors of [72] employed an ML approach to GPS jamming detection. A testbed was developed to generate synthetic GPS signals utilizing GNU Radio with a National Instruments B-210 Universal Software Radio Peripheral (USRP) both with and without the presence of a variety of types of jammer signals that were fed to a u-blox M8 GPS receiver. Four types of jamming signals were considered: barrage noise, single tone, successive pulse, and protocol-aware (P-aware). The authors then performed feature analysis to determine the most salient features in the resulting signal data, such as position accuracy, HDOP, and COP, and used those features for each dataset to train various types of ML models. The performance metrics included detection rate (DR), misdetection rate (MDR), false alarm rate (FAR), and F-score (FS). The authors established that a neural network approach yielded the best performance, with DR, MDR, FAR, and FS values of 98.9%, 1.39%, 0.28%, and 0.989, respectively. K-Nearest Neighbor (KNN) yielded similar performance but suffered from much longer prediction times. Random Forest approaches also performed well but experienced longer prediction times. Other approaches, such as Decision Tree (DT) and Support Vector Machine (SVM), yielded poor performance. This neural network approach is promising and may eventually yield a good, generalized solution to GPS jamming detection; however, an unanswered question is how this approach will perform in complex propagation environments, where the GPS signal and corresponding positioning quality may be rapidly fluctuating. Furthermore, it is unclear if the computational requirements for this type of approach are compatible with embedded applications. 

### 5.2. GPS Spoofing Detection

GPS spoofing has been a recent topic of intense interest; therefore, there are many published papers on the subject. There are three primary approaches for GPS spoofing detection: ML-based, antenna/direction-of-arrival (DOA)-based, and movement tracking-based (Table 10).

Most of the proposed methods for GPS spoofing detection are based on machine learning approaches. These approaches share a largely common workflow: (1) conduct a set of experiments for which the outputs are known; (2) measure a set of observable parameters that have been deemed essential; (3) compile those measured values along with the known outputs into a training dataset; (4) train the machine learning model; (5) evaluate the performance of that machine learning model with a separate dataset of measured parameters and known outputs; and (6) compare the outputs predicted by the machine learning model with the known outputs. The key differences between the proposed approaches are (1) features chosen for inclusion in model training, (2) model selection, (3) chosen performance metrics, and (4) achieved performance. Table 11 summarizes some of the proposed ML-based approaches.

For instance, the authors of [84] proposed an SVM-based approach to GPS spoofing detection, where they looked at the difference between position as derived from GPS and the onboard inertial sensors and then determined if the difference was due to spoofing or inertial errors through an SVM model. The authors of [85] presented a linear regression approach to GPS spoofing detection. In this approach, the UAS flight trajectory prediction model was obtained by fitting the UAS’s flight log with the linear regression model. The authors of [86] proposed using fuzzy logic in the signal acquisition process. The proposed approach aligns the value of the acquisition threshold using parameters affecting acquisition performance in the presence of the spoofed GPS signal. The ratio between the correlation levels was then used to distinguish between the actual and spoofed GPS signals. The authors of [87] proposed a game-theoretic approach to GPS spoofing detection for UAS applications. The proposed approach viewed the interactions between a GPS spoofer and UAS operator as a Stackelberg game, showing this approach outperformed other game strategies.

ML-based approaches are the current most popular method; however, a wide variety of existing approaches are not ML-based. These approaches can be classified as: (1) antenna-based approaches; (2) movement history-based approaches; and (3) signal statistics-based approaches. Many other proposed approaches do not easily fit into any generalized category or taxonomy.

The authors of [88] proposed a microstrip patch antenna array-based solution that determines the Direction of Arrival (DOA) of the spoofed GPS signal through a DL-based approach. The DOA of authentic GPS signals is roughly known (upward-facing hemisphere); therefore, signals that are determined to have DOA values outside of that plausible range are deemed spoofed signals. The performance of the proposed approach is a function of three parameters: (1) the SNR of the actual GPS signal, (2) the SNR of the spoofed GPS signal, and (3) the number of antenna array elements. Good performance is achieved with a sufficiently high GPS SNR: greater than −4 dB relative to the spoofed signal. The best performance was observed when the number of antenna array elements was greater than six.

Similarly, the authors of [89] proposed an approach to detecting GPS spoofing by determining the DOA of the spoofed signal. The authors of [89] proposed using a compressed sensing method instead of a DL method to estimate the power and DOA of the incoming signal based on off-grid Bayesian inference. Simulation results demonstrated the ability to accurately estimate DOA towards the GPS spoofer when the power of the received spoofed signal was greater than that of the actual GPS signal and when the DOA of the spoofed signal was different from that of the actual GPS satellites. The authors of [90] proposed using a dual-antenna system to calculate the Doppler Frequency Difference of Arrival (FDOA). The approach is predicated on the consistent, predictable nature of the GPSS downlink signal and the fact the presence of a spoofed GPS signal can be determined by detecting subtle differences in carrier frequency and phase, demonstrating that the proposed approach can discern between the actual and spoofed GPS signals. The advantage of this approach is its computational simplicity, since it requires little memory or processing capability. The disadvantage of this approach is the need for multiple antennas that must stay in the same formation during the observation window, making it unclear how well this approach would perform in the case of platform mobility. Another antenna array-based method was proposed by the authors of [91], in which the antenna array would be used to detect spoofed signals based on phase delay measurements. The primary drawback to these approaches is the need for multiple antennas, which may not be practical for small systems.

Other researchers proposed using approaches based on tracking the UAS movement history for GPS spoofing detection. For example, the authors of [92] proposed a multi-sensor approach that combined the onboard IMU sensor with vision methods using a monocular camera onboard the UAS. The proposed approach would use the camera’s video stream and IMU sensor to calculate a velocity vector for the UAS. The video feed would be used to estimate platform velocity, and that information would be used to reset IMU error accumulation. Simultaneously, a velocity vector would be calculated based on GPS alone. Those two velocity vectors would be compared, and spoofing would be declared if they were sufficiently different. This approach was implemented on a DJI Phantom 4 UAS and could be detected within an average of five seconds. The authors of [93] also proposed using IMU sensors to detect GPS spoofing attacks. This approach was implemented by integrating an IMU/GNSS into a Kalman filter that monitors anomalies.

Several papers exist in the literature that analyze various aspects of signal statistics, of both the authentic signal and the spoofed signal, in detecting the presence of a spoofing threat. As an example, the authors of [94] considered the use of Doppler frequency and carrier-to-noise (C/N) density ratio as the primary metrics to discern a spoofed signal from an authentic signal. The authors then used numerous types of ML and analytical models to analyze performance utilizing these metrics, including SVM, KNN, RF, Gradient Boosting Decision Tree (GBDT), DT, and XGBoost. The authors of [94] reported accurate spoofing detection rates of 84.88–95.56%, with KNN providing the best results in terms of accuracy for their datasets. SVM provided nearly as good accuracy, with slightly better true positive rate (TPR) performance.

Many other existing approaches do not cleanly fit into a taxonomy. The authors of [95] proposed an approach to GPS spoofing detection that treats GPS spoofing detection as an outlier detection problem, analyzing the time-series history of the position estimates and identifying outlier values through the Grubbs outlier test. Any outlier is a spoofed position estimation in this approach. The authors of [96] also treated GPS spoofing as an outlier problem. They implemented a navigation filter based on a constant velocity model. This model employed a 3D Kalman filter to identify and remove outlier values, which were presumed to be spoofed locations. The authors of [97] also viewed GPS spoofing detection (in part) as an outlier detection problem, employing a Kullback-Leibler divergence measure to search for anomalies in data. The authors coupled this anomaly detection approach with an entropy-based method for spoofing detection. The outstanding question regarding outlier removal approaches is how they would work in dynamic mobile scenarios that do not have a constant velocity model or where signal fading causes rapid signal power fluctuations, both actual and spoofed GPS signals.

Other proposed approaches utilize ADS-B broadcasts from aircraft and UASs to detect GPS spoofing attacks. Ground sensors monitor ADS-B broadcasts while geolocating the corresponding aircraft. It is assumed that the aircraft is a victim of GPS spoofing if the ADS-B broadcast reflects a different position than its actual position. The authors of [98] claimed that this approach can globally detect GPS spoofing attacks in under two minutes, and they can localize the attacker with an accuracy of 150 m within 15 min of monitoring time. The authors of [99] claimed an average detection accuracy and precision of 81.7% and 85.3%, respectively, based on real-world air traffic control (ATC) data crowdsourced by the OpenSky Network. A potential limitation of these approaches is that, like GPS, ADS-B broadcasts are unauthenticated and can be spoofed.

The authors of [100] utilized a satellite imagery matching technique to detect GPS spoofing attacks in UAS applications. In this approach, DeepSIM uses the onboard camera to capture images of the terrain below and then compare those to existing satellite-based imagery to determine its position, which can then be compared with the position determined via GPS. A discrepancy between the two is illustrative of GPS spoofing. The authors utilized four different DL models to achieve image matching based on whether satellite imagery or aerial imagery was available for the area, including Distance Threshold, Siamese ResNet, Semi-Siamese Network, and Siamese SqueezeNet. The authors reported a GPS spoofing detection rate of over 95% using this approach. A potential limitation of this type of approach is the ability to capture all possible conditions in the DL training datasets to reflect different light conditions (e.g., night versus day), changes in seasons (e.g., foliage changes), manmade or natural-induced changes to terrain, etc.

## 6. Countermeasures and Their Comparison

### 6.1. Countermeasures for GPS Jamming

There are limited options to mitigate the effects of interference once it is detected. Mitigations to GPS interference, either intentional or unintentional, primarily focus on the rejection of the interfering signal, minimizing its effect on the GPS receiver. This interference rejection is achieved primarily through antenna-based approaches, such as antenna nulling, or filtering, such as notch filtering the interference.

#### 6.1.1. Antenna-Based Approaches

These approaches are predicated on antenna arrays that can be electronically steered. A null in the received antenna pattern can be formed once the interference signal has been detected, and the power of the interference is reduced while maintaining the power of the actual GPS signal. Many papers in the literature propose an antenna-based approach to mitigate the effects of GPS jamming. These approaches involved complete solutions that included interference detection methods and countermeasures. The factors that differentiated these various proposals were (1) antenna technology, (2) measured signal attributes used for antenna null formation, (3) speed of adaptation, and (4) resultant performance, such as depth of nulls. Many other papers proposed anti-jam GPS antenna designs but did not propose interference detection methods or antenna adaptation algorithms. These papers typically assume their proposed antenna design can be paired with suitable adaptation algorithms and measurement approaches. Other studies presented antenna adaptation algorithms but did not present either detection methods or actual antenna design. Many antenna-based approaches previously discussed for GPS jamming and spoofing detection also apply to GPS jamming mitigation. Table 12 summarizes some of the antenna-based approaches found in the literature. 

The question surrounding antenna-based approaches is whether these designs and techniques can be made sufficiently small and simple enough to be feasible options for small systems. Microstrip antennas may be more attractive for UASs than larger arrays; however, small arrays with dipole antenna elements could be feasible, especially for larger unmanned platforms. None of these techniques are likely feasible for smaller unmanned platforms, such as micro UASs.

#### 6.1.2. Signal Processing Approaches

Several approaches exist in the literature based on reducing/removing unwanted interference through signal processing methods, focusing on some form of notch filtering, some of which are summarized in Table 13.

Other research papers have studied the effects of various hardware components on jamming rejection performance, such as the automatic gain control (AGC) function within the GPS receiver [109], in which they performed frequency domain analysis to isolate the desired GPS signal from the jamming signal. This frequency domain method can effectively capture GPS signals and begin processing when the AGC interference-to-noise ratio is at least 37 dB, corresponding to a GPS SNR of approximately 14 dB. These signal-processing approaches are typically effective against only specific types of jamming waveforms; however, they are relatively lightweight in terms of computational or hardware complexity and are likely feasible across many hardware platforms.

Most proposed techniques primarily address narrowband jammer types, such as CW and narrowband chirp jamming. Limited signal processing-based approaches in the literature address wideband jamming threats. Wideband jamming techniques are generally less effective than narrowband techniques, likely due to the spread spectrum nature of the GSM waveform, suggesting that the lack of wideband methods is not a critical gap; however, studies have indicated that partial-band swept noise can be highly effective in disrupting GPS signal reception. Current literature does not address mitigating this type of jamming.

### 6.2. Countermeasures for Spoofing

Many antenna-based approaches for jamming mitigation can also be applied for GPS spoofing mitigation. Many of these approaches are predicated on determining the DOA of the interfering signal and then creating a null in the receive antenna pattern in the direction of the hostile attack system. Most of these approaches, particularly those that work on the carrier phase, can also be applied to the spoofing threat. Additional papers in the literature presented antenna-based approaches that optimized their detection and antenna nulling specifically to address GPS spoofing by taking advantage of code-based GPS signal structure features. For example, the authors of [91] looked at carrier phase differences in received signals by taking advantage of C/A code symbol alignment properties between the actual and spoofed GPS signals, reporting an attenuation of unwanted signals of at least 60 dB when the SNR of the spoofed signal was high. Most antenna-based methods discussed for GPS spoofing detection also include mitigation approaches focused on creating antenna nulls in the direction of the GPS spoofer. Most signal processing-based approaches to GPS jamming mitigation do not apply to GPS spoofing mitigation. Most papers that propose signal processing-based jamming mitigation approaches primarily address narrowband unwanted signals and cannot remove the wideband spoofed GPS signal.

The common approach to GPS spoofing mitigation is simple message filtering. GPS spoofing detection methods focus on identifying the presence of the spoofed GPS signal. The assumed mitigation approach is for the system to simply ignore the resulting position once it is detected. This capability is essential since the unmanned system will no longer follow false position estimates and can no longer have its flight path manipulated by the GPS spoofer. 

### 6.3. Countermeasures for GPS-Denied Environment—Alternate Positioning

**Figure 5:**
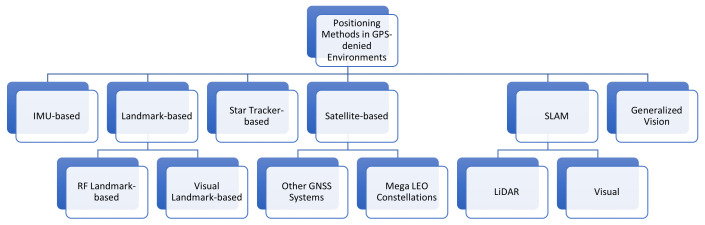
High-Level taxonomy of existing methods for positioning in GPS-denied environments.

Researchers have proposed many techniques for positioning and navigation in GPS-denied environments over the past two decades [23]. These different approaches are all predicated upon using measurements from onboard sensors to understand its environment, from which position can be inferred. Different approaches utilize different sensors, while some utilize values from multiple sensors. These proposed approaches generally belong to one of six categories (Figure 5) [110].

Not all systems will possess all sensor types. Consequently, not all alternate positioning methods will universally apply to all unmanned systems. Furthermore, these various approaches have strengths and weaknesses that may make them more applicable to certain use cases. The advantages and disadvantages of these various alternative positioning approaches are summarized in Table 14 [110].

#### 6.3.1. IMU-Based Approaches

IMUs are packaged sensor suites that typically include accelerometers and gyroscopes for each axis of motion and sometimes contain magnetometer sensors. An accelerometer is an instrument that measures linear and angular acceleration, such as changes in speed or direction. Accelerometers can be mechanical, capacitive, or piezoelectric in nature. A gyroscope is a device used to measure or maintain orientation and angular velocity. A magnetometer is a device that measures magnetic field strength, allowing the UAS to always know magnetic north, similar in function to a compass. IMU sensors measure acceleration, orientation, angular rates, and magnetic and gravitational forces. These measurements can then be used to detect and track motion relative to the system’s starting point, as in determining relative position and velocity [23]. IMU-based approaches are very mature and can provide highly accurate short-term positioning and navigation results; however, IMU accuracy decreases over time due to inherent error accumulation. These errors can take the form of biases, scale-factor errors, and misalignment errors, leading to significant inaccuracies after as little as one minute of use. IMU calibration procedures can significantly lower these errors; however, some level of error accumulation will generally linger, which will eventually yield inaccurate results. For example, magnetometer sensors often experience noisy measurements due to the strong currents in the electric motor circuits during flight, such as motors turning rotor blades in a quadcopter. Research in the literature provides recommendations for proper ground calibration to minimize this noise [111]. Another common approach is employing extended Kalman filters (EKFs) to dynamically weight IMU sensor measurements, which can further reduce accumulation errors and unwanted biases.

Quantum accelerometers are an exciting research area that has the potential to eventually provide very good IMU-based positioning and navigation, primarily due to the extremely low errors these quantum devices produce, meaning that error accumulation would be insignificant; however, quantum accelerometers do not yet exist in a practical form for most applications. Quantum accelerometer technology faces many challenges, making the technology impractical for most platforms. Consequently, researchers are actively searching for methods to improve IMU performance. The authors of [112] have established that the key technical challenges facing quantum accelerometers are (1) a lower sample rate due to cold atom interrogation time and (2) a reduced dynamic range due to signal phase wrapping. 

Numerous papers in the literature focus on improving IMU performance for positioning and navigation. Two key trends in the literature propose IMU-based approaches: (1) IMU redundancy and (2) fusion of IMU with other onboard sensors into multi-fusion ensemble approaches. 

The authors of [113] proposed an approach that employs an array of six commercial-grade inertial sensors arranged in a cube containing triaxial gyros, accelerometers, and magnetometers. This approach aims to compensate for individual sensor bias using mutual calibration. This mutual calibration is accomplished by calculating average values across all sensors for each spatial axis. This approach was demonstrated in a vehicle-based experimental testbed with reported average position accuracy within 1.1 m. The multi-IMU approach outperformed GPS-based positioning during experimentation, providing an average position accuracy within 3.52 m. The authors of [113] continued their work in [114], focusing on the accuracy of attitude determination in UAS applications. They again used their multi-IMU system with six IMUs in a cubic configuration and focused on assessing the accuracy of roll, pitch, and heading. The reported root mean square error (RMSE) was extremely low, with heading, pitch, and roll errors reported as 0.032, 0.012, and 0.023 radians, respectively. The authors of [115] proposed a multi-IMU redundancy approach, where they fused measurements from five different IMUs using a feedback-federated Kalman filter with attitude estimation using the Bortz equation, resulting in the best performance of all methods considered. This approach achieved very low errors in estimating the test platform’s roll, pitch, and yaw compared with ground truth. These various research efforts established the feasibility of using multi-IMU approaches in terms of achievable performance. The major challenge with these approaches is the higher cost and required sensor hardware, which may be prohibitive for some systems.

The research presented in these papers has one primary limitation: they all evaluate their solutions in well-controlled vehicle-based or small UAS-based systems in seemingly benign environments. One main limitation of IMUs is that inertial sensors are extremely sensitive to environmental and platform factors, such as temperature, pressure, mechanical vibration, and electrical system noise, in the case of magnetometers. Many unmanned systems will be expected to operate in harsh environments that may produce fluctuations in these parameters and significant system vibration, which complicates effective IMU calibration. The mutual self-calibration approach proposed in [113] is promising since it may solve this problem; however, additional experiments and analyses are required to determine performance under harsh conditions.

Most IMU-related research focuses on multi-sensor fusion and using additional sensors to improve IMU-based positioning performance. The underlying concept of these approaches is that IMUs can provide very accurate positioning solutions for limited periods of time due to error accumulation. Additional sensors are used to periodically achieve an accurate ground-truth position estimate that can be used to reset the IMU-based method. The IMU-based method then needs to simply maintain accuracy until the next ground-truth solution can be achieved via the other sensors. A common approach is using a hybrid IMU-GNSS approach with GPS-provided ground truth to reset inertial accumulation errors, with the IMU maintaining positioning during GPS intermittent outages. This approach does not work in the GPS-denied environment; another sensor is required to provide this ground-truth IMU reset function.

The authors of [116] proposed an inertial navigation algorithm that incorporates sensor outputs from accelerometers, gyroscopes, magnetometers, Pitot tubes, and air vanes; however, a stated goal of their approach was to facilitate the fusion of their proposed inertial filter with visual odometry methods. Several papers have proposed using magnetometer sensor outputs to improve IMU performance. The authors of [117] proposed to fuse inertial and magnetometer sensors using a Kalman filter to improve performance. The authors of [118] proposed an integration of IMU outputs with magnetometer sensor outputs to improve accuracy in attitude initialization. Numerous papers have proposed fusing IMU-based techniques with various sensors that may be onboard the UAS, including vision techniques utilizing an onboard monocular camera [119], altitude estimation using a range sensor [120], SLAM techniques using LiDAR [121], and LEO satellite tracking [122]. Several research papers also propose a joint IMU-GPS positioning solution to improve performance where GPS reception is intermittent or inaccurate, such as in urban environments [43,123]. These papers report low-achieving position estimation errors, generally within 10 m. The primary disadvantage of these proposed methods is the need to include additional sensors onboard the platform.

Few studies have proposed ML-based approaches for nonlinear processing to minimize inertial drift. For example, the authors of [124] proposed a method where IMU sensor output error would be estimated and corrected by employing a non-linear auto-regressive neural network with exogenous inputs cascaded with a multi-layer perceptron (MLP)-based neural network. The proposed approach outperformed recurrent neural network (RNN)-based and legacy EKF approaches. The authors of [124] reported position estimation accuracy improvements over tactical-grade IMUs of 30%, 44%, and 80% for GPS outages of 10, 25, and 50 s. These results are encouraging; however, error drift still limits the useful timeframe for navigation based on IMUs alone.

Redundant IMU and multi-sensor fusion methods offer good performance with a low cost of computational complexity. The major disadvantage of these approaches is the need for additional sensor hardware onboard the UAS, which may be problematic for small and low-cost systems; however, there are examples of multi-sensor fusion approaches working on small micro-UASs. The authors of [125] demonstrated a multi-sensor fusion navigation system onboard a micro-UAS that utilized 3D LiDAR, a stereo camera, an altimeter, and IMU sensors, with data fused using a synchronization and time delay compensation algorithmic strategy. This research demonstrated that these multi-sensor approaches are achievable on small UAS platforms.

A technical challenge not addressed in the literature is the initial determination of the ground-truth position. IMU-based methods provide relative position information and must have an accurate ground truth from which to start. Onboard sensors may prove less useful in providing this ground truth. This problem may require manual intervention if the unmanned system is initiated from a well-known location.

There have been proposed methods that rely solely on magnetometer sensors for navigation [126]. The MagNav concept uses Earth’s magnetic field to uniquely identify any point globally. Good performance has been observed using MagNav approaches, reaching the destination of a 1500-mile flight to within 1 km of accuracy using nothing but a magnetometer and machine learning algorithms trained on magnetic field map data. A significant challenge with this approach is that Earth’s magnetic field is constantly changing, requiring periodic magnetic field surveying and model retraining.

#### 6.3.2. Landmark-Based Approaches

Many techniques for positioning and navigation in the literature are based on the idea of detecting known objects within the environment, such as landmarks. These approaches utilize the known position of landmark objects so that the UAS knows its location when that object is encountered. The logic behind this concept can be best explained with this illustration: I know where that thing is located. I see that thing. By extension, I know where I am. We have already seen variations of these approaches in the IMU approach discussions. Other sensors are used to locate recognizable objects in the multi-sensor fusion IMU approach, establishing a ground-truth position estimate that can be used to reset IMU accuracy. The landmark concept is identical, except an IMU is not necessarily present. Instead, position and navigation may be achieved solely through landmark recognition. Landmark recognition may also be used intermittently in conjunction with other sensors, such as an IMU. Landmark-based positioning requires the UAS to maintain knowledge of known object locations so that when that object is encountered, it can be mapped to a location. The “object” can be any phenomenon that can be observed via a UAS sensor. The existing ones commonly fall into one of two categories: (1) RF landmarks and (2) visual landmarks.

RF landmark-based approaches use existing communications infrastructure for position calculation, which could take the form of signal emitters pre-placed along the UAS flight path. This approach requires a priori knowledge of the flight path and the installation of ground-based infrastructure. These types of approaches have been effective in the case of visual landmarks [127] and also perform well in the case of RF landmarks [128]. This pre-placement approach may prove valuable in applications where a UAS always performs a fixed flight path; however, it may not be practical for generalized UAS operations.

A more flexible approach is utilizing existing commercial communications infrastructure for position calculation. In this case, existing commercial signals are viewed as Signals of Opportunity (SOPs) that are received by the UAS platform. The locations of many types of commercial RF emitters are publicly known; therefore, it would be possible to build an onboard emitter location database that could be used to detect SOPs and estimate the receiver’s position. There are many papers in the literature where commercial SOPs are used for navigation purposes, including IEEE 802.11-based Wi-Fi [129], Bluetooth [130], AM Radio [131], FM Radio [132,133], Digital Television (DTV) [134], and cellular communications [135]. The authors of [136] implemented an SDR-based system onboard a small quadcopter UAS using FM radio, DTV, cellular, and Wi-Fi SOPs for positioning, demonstrating good position accuracy performance. It is unclear how SOPs such as Wi-Fi and Bluetooth could be incorporated into a generalized positioning system since Wi-Fi and Bluetooth locations are not always publicly known; however, many of the other SOPs, including AM radio, FM radio, DTV, and cellular infrastructure, lend themselves to a generalized approach since their emitter locations are well known and publicly available. Many technical attributes of these emitters, such as transmit power and antenna type, are also publicly available.

One limitation of this approach is that it will not necessarily be applicable across all geographic locations. Many papers in the literature focus on urban and suburban regions; however, there will be a less dense communications infrastructure for many of these signal types, such as DTV, in rural and less populated regions. A promising approach for positioning is using signals from the commercial cellular communications infrastructure as SOPs. Previous studies have revealed that good accuracy can be obtained; however, accuracy depends on the environment, and position accuracy is sensitive to interference [135]. Numerous studies in the literature demonstrate that good position accuracy can be obtained using 4G LTE cellular SOPs [135,137,138,139,140,141]. There is also a growing amount of literature demonstrating that good performance can be obtained using 5G cellular SOPs [142,143,144]. Previous work has demonstrated accurate positioning using CDMA-based cellular SOPs [145].

Cellular SOPs could be cooperative or uncooperative. A cooperative SOP paradigm means that the system is a participating member of the cellular network. This approach allows the User Equipment (UE) to receive positioning information from the cellular network via its existing positioning method, such as the LTE Positioning Protocol (LPP) in 4G cellular networks. Cellular UE positioning via these built-in positioning methods and the high accuracy of these methods are well understood; however, research has suggested that the performance of these methods may be sensitive to jamming and interference [143]. This approach may not work in regions without significant cellular infrastructure, such as unpopulated desert regions. There are also potential negative impacts on the overall cellular network associated with UAS-based UEs. This issue is due to the increased visibility of the cellular network by the UAS-based UE, potentially increased uplink interference, and a higher number of system handoffs [146]. The second approach is uncooperative in nature, where the system is equipped with an RF receiver capable of passively observing cellular signals and using them as landmarks but is not a valid user of the cellular network.

A fundamental concern with RF-landmark-based approaches is that these signals are all unauthenticated, and while publicly available information is valuable in developing an onboard knowledge base, it is also valuable to someone launching a malicious attack. It is feasible that someone could mount an attack where they not only spoof GPS but also any of the signals mentioned above since SDR technology is easy to obtain due to its convenience and low cost. This aspect of RF-landmark research has not been addressed in the literature to date.

Visual landmark-based approaches are predicated on detecting landmarks in imagery captured via a camera sensor. These landmarks can be either manmade or natural and, combined with some type of onboard knowledge base, could be used to determine position with high demonstrated accuracy [127,147]. An example of landmark preplacement for visual recognition in support of navigation can be found in [148], where landmarks are strategically established in a partially known environment to support robot navigation. Another example can be found in [149], where the authors used a monocular camera onboard a robotic ground vehicle to detect pre-placed landmarks that served as waypoint markers. The generalized applicability of these approaches is limited due to their reliance on well-known existing landmarks or pre-placed objects along the path of movement; however, these approaches may be useful when operating in known areas or fixed flight paths. These types of approaches could also be helpful in hybrid approaches. Combining visual landmark-based approaches with IMU-based approaches can achieve high position estimation accuracy [115], where visual recognition provides intermittent high-accuracy ground-truth position estimates that can be used for IMU accumulation error reset. The authors of [115,127] demonstrated that this type of approach can work well within sufficiently dense regions with visually identifiable landmarks. For example, consider using roads as landmarks, where a UAS equipped with a monocular camera detects roads within images taken from the camera and recognizes the ground road pattern to determine its own position. This approach has been proposed by multiple papers in the literature [150,151], with good position accuracy performance reported; however, landmark-based approaches do not perform well in feature-sparse regions, such as deserts and oceans. Furthermore, many of these approaches rely on ML-based neural network approaches that will place a computational requirement on the UAS.

There are several technical challenges associated with visual landmark-based navigation. These methods are typically computationally expensive and may not be suitable for many unmanned systems; visual methods generally do not perform well in low-light scenarios, which may limit the scenarios in which this method may perform well; and visual methods do not perform well when reference scenery or objects change. Causes of change can be manmade, such as in the presence of construction, or natural, such as changes in seasons or time of day.

#### 6.3.3. Star Tracker-Based Approaches

A star tracker is a sensor that recognizes star patterns in the sky and can be used for navigation purposes. Star tracker-based navigation approaches have existed for thousands of years, particularly in nautical applications. Modern star tracker systems are mature technology. Star tracker systems are commonly used in space-based and aviation-based navigation applications; however, star trackers by themselves do not typically provide position estimates. Rather, they are used to determine orientation and can be used in conjunction with other sensors to determine position. Star tracker methods can achieve high accuracy in orientation determination. These systems perform well in low-light and clear-sky scenarios; however, their performance suffers in daylight hours due to interference from the sun. Performance also suffers in cloudy conditions due to the occlusion of the observable stars.

Researchers have recently attempted to leverage star tracker techniques and improve performance, although this is not an active research area. There are examples of researchers attempting to fuse star tracker data with IMU data in a multi-sensor fusion approach [152,153]. There are also examples of ongoing work to attempt to improve star tracker performance during daytime operations [154,155]. The authors of [155] proposed an approach where they characterized ambient polarized skylight and then fused skylight, starlight, and IMU data through a Kalman filter approach. The authors of [154] focused on characterizing sky background radiation and stellar radiation, which can be used to calibrate and configure the star tracker hardware and software for optimal detection. The results from these papers are promising; however, star tracker performance in the daytime continues to be a significant limitation and an unsolved problem. Star tracker systems are often expensive, limiting their applicability to many unmanned systems. This research area is unlikely to experience near-term technical breakthroughs since active research is limited.

#### 6.3.4. Satellite-Based Approaches

There is a growing amount of research on using other satellite systems to augment GPS-based navigation, of which there are two primary categories of approaches: (1) multi-GNSS approaches and (2) commercial LEO constellation-based approaches, such as mega-constellations. Commercial multi-GNSS chipsets can be configured to receive signals from GPS, GLONASS (Russia), Galileo (European Union), and BeiDou (China). Multi-GNSS chipsets inherently mitigate the threat of attack on any one GNSS, with a limited number of papers in the literature that quantitatively support this claim. The authors of [156,157] conducted analyses and experimentation with multi-GNSS receivers to characterize their performance, establishing that multi-GNSS approaches can outperform any single GNSS system. The authors of [157] analyzed the performance of multi-GNSS receivers in the presence of jamming and established that position accuracy is largely unaffected due to the availability of other GNSS systems, even if a single GNSS signal is made unavailable. The authors of [158,159] proposed methods for automatically selecting which GNSS to use for position estimation based on observed conditions. The authors of [159] proposed a method to treat all GNSS constellations as sub-systems of a single system and then selectively choose which satellites from each constellation would be used for position estimation. These papers all demonstrated high position estimation accuracy, as expected, with errors typically of five meters or less. It is intuitively obvious that a multi-GNSS receiver will generally outperform a dedicated GNSS receiver; however, this approach has one key limitation. Other GNSS systems, such as GPS, are susceptible to jamming and spoofing attacks. Consequently, these systems cannot necessarily be trusted any more than GPS. The diversity of employing multiple GNSS systems will improve system robustness; however, an adversary capable and willing to launch an attack on GPS is likely willing and able to do the same against all the other systems. Developing a multi-GNSS jamming or spoofing attack with the SDR-based attack systems found in the literature would be relatively straightforward.

There is an increasing amount of research in the literature that is focused on the use of non-GNSS satellite systems such as Starlink, OneWeb, OrbComm, and Iridium for navigation [160], with Starlink receiving the most interest in the literature to date. These constellations are extremely large (thousands of satellites) and very fast-moving from the perspective of a ground-based observer due to their Low Earth Orbit (LEO) positions, which largely mitigates a physical attack scenario. Much of the work in the literature has been performed by Kassas and his research team, the authors of [160] and numerous other papers on the subject [161,162,163,164,165]. The authors investigated using numerous features of the downlink signals from these constellations, with a focus on Starlink. Some of the more promising signal characteristics are carrier phase and carrier Doppler shift.

Other research teams have recently begun studying the use of these commercial mega-constellations for navigation purposes [166,167,168,169]. Many of the proposed methods would require modification to the downlink signal of these commercial systems, which is unlikely to happen; however, many other proposed approaches are completely passive and utilize the as-is downlink signals. The results presented in these papers are promising and demonstrate that this is a feasible concept; however, the downlink signals from these systems will likely be susceptible to jamming or spoofing attacks, especially given the capability and flexibility of SDR-based GPS attack systems.

#### 6.3.5. SLAM Approaches

Simultaneous Localization and Mapping (SLAM) is a special category of navigation and positioning that aims to characterize and navigate a local GPS-denied environment via its onboard sensors. SLAM methods generally fall into three categories:

(1) Light Detection and Ranging (LIDAR)-based SLAM [170,171,172,173,174,175,176,177];
(2) Visual-SLAM (vSLAM) [178,179,180,181,182,183,184,185,186,187,188,189,190];
(3) Hybrid Visual-LiDAR SLAM [191,192].

These SLAM approaches typically consider the scenario of an unmanned vehicle navigating a localized region, such as a small UAS exploring an underground cave system. These approaches can be either map-based, where the UAS is attempting to navigate an area from a known map, or mapless, where the unmanned system is attempting to navigate an area while simultaneously creating a map of the region. In both cases, the unmanned system attempts to detect features in the environment and use those features to determine its location. LiDAR-SLAM approaches detect regional features via LiDAR sensors. A primary limitation of LiDAR is cost, since LiDAR sensors are expensive compared with other sensor types, limiting their applicability. vSLAM approaches use the onboard camera sensor. Most vSLAM techniques propose monocular camera sensors that serve vSLAM or VO purposes [193,194,195]. A primary limitation of vSLAM is its computational complexity, which also limits its applicability. There is a relatively even mixture of 2D versus 3D LiDAR-SLAM papers in the literature; however, 2D LiDAR-SLAM is far more mature. Most 3D LiDAR-SLAM papers focus on finding solutions to the various challenges of 3D SLAM.

These approaches are mature and have demonstrated their ability to map and navigate highly complex environments, including obstacle avoidance; however, they typically do not provide absolute positioning solutions. SLAM methods may not directly apply to generalized positioning and navigation; however, much of the research within the SLAM community is directly applicable to approaches for generalized positioning. For example, significant research within the vSLAM community is focused on improving object and feature detection performance in low-light conditions, which is also a limitation within the generalized vision approaches to positioning, and lessons learned across these communities will benefit both groups.

#### 6.3.6. Generalized Vision Approaches

vSLAM methods typically operate in specific regions and do not necessarily provide absolute positioning; however, generalized vision-based approaches attempt to provide generalized absolute position estimates. Landmark-based vision approaches attempt to determine position based on simple landmarks; however, generalized vision approaches aim to operate on general terrain imagery to provide absolute positioning, including using latitude and longitude. Consider the case of a UAS-based system, where a UAS-based camera sensor observes the ground and an onboard neural network-based algorithm performs terrain recognition to determine the system’s position. The authors of [196] proposed an approach where camera imagery was matched to pre-existing aerial satellite imagery to determine location. Factors such as weather, lighting conditions, and seasonal changes to terrain led to poor performance. A positioning accuracy of 36.4 m was achieved using a contrastive learning approach by implementing a convolutional neural network (CNN)-based Siamese neural network that then compared imagery from the UAS camera to aerial satellite imagery. A similar approach was proposed by the authors of [197], who utilized a CNN trained with a large set of preexisting satellite imagery to predict location based on ground images from the UAS camera. The authors of [197] combated issues such as different lighting conditions, weather, and seasonal changes by building a large training dataset that contained images representative of all these conditions. The authors of [110] proposed using the You Only Look Once (YOLO) object recognition algorithm trained on aerial imagery. The trained YOLO model was then used to evaluate imagery from the onboard camera, demonstrating approximately 50-m position estimation resolution with a limited training dataset size; however, the approach performed poorly in regions sparse in features, such as a desert environment. Another interesting approach was proposed by the authors of [198], who built and trained an ML model with images representing each ground coordinate, like the proposed approach in [197]; however, they built the training dataset using Digital Elevation Map (DEM) images for each corresponding location instead of actual images. The goal was to then perform basic image processing on the image from the camera and then input that image into a CNN model for detection and classification. This approach was more immune to changing light conditions and seasonal changes than traditional image-based approaches.

A key technical challenge with generalized vision approaches is the complexity of training the deep learning algorithms typically associated with these approaches. Consider the analysis of this issue presented in [110], which illustrates the dataset size requirements for a single global training dataset sufficiently representative of all locations on Earth. A globally applicable dataset that can achieve 10-m position accuracy resolution would require a minimum dataset size of approximately 1.5 × 10^15^ images, assuming a single image representing each latitude and longitude coordinate separated by 10 m. These extremely large image training dataset sizes are problematic due to the corresponding computational requirements for model training. The authors of [110] proposed a region-based visual position estimation method with other sensors providing a coarse position estimation, in this case, an RF-based sensor utilizing cellular infrastructure, to mitigate this computational requirement. The resultant coarse position estimate was used to apply a region-specific visual method for precise position estimation selectively. These region-specific visual algorithms were trained against much smaller training datasets specific to a region, reducing computational complexity.

## 7. Research Gaps, Challenges, and Future Research Directions

Detecting and mitigating threats to the GPS has recently received significant attention. This paper provides an overview of the environments and attacks in which GPS performance may be degraded or lost. GPS jamming and spoofing attacks are becoming increasingly common, and systems capable of executing these attacks can be readily achieved with low- and no-cost commercial hardware and software components with minimal expertise. The data available in the literature illustrate the commonality of GPS jamming attacks worldwide. Unfortunately, no real-world data were found available for GPS spoofing attacks. A useful measurement campaign would be to establish long-term GPS monitoring sites and pair these sites with some of the advanced GPS spoofing detection approaches found in the literature to determine the frequency of GPS spoofing attacks.

Numerous papers in the literature characterize GPS performance. Some of these papers illustrated the performance challenges of GPS in complex multipath and shadowing propagation environments. Some of these papers illustrated the variability in performance across different commercial GPS receivers; however, most of the experiments presented in these papers were not conducted in a controlled manner or with reported rigor. These papers gave a sense of the challenges and performance variability; however, the experimentation methods in most of these papers unfortunately mean that no definitive quantitative conclusions can be drawn from them. Instead, high-level qualitative trends can be inferred from these papers. There are two useful future research activities in this area: (1) controlled and exhaustive experimentation to develop detailed quantitative models of GPS performance variability across GPS receivers, and (2) controlled experimentation to develop detailed channel models for GPS propagation in complex multipath fading and signal shadowing environments. Furthermore, there is a need for additional research to characterize the performance of GPS receivers in the presence of jamming, such as chirp jamming and partial-band noise jamming.

There are a limited number of papers about GPS jamming detection. Most papers focused on two approaches: (1) antenna-based and (2) signal statistics-based. Many of the antenna approaches are promising, but they require antenna arrays that are too large and complex for UAS platforms. Most of the signal statistics-based approaches make simplistic assumptions regarding the propagation environment, and consequently, it is unclear if many of them would perform well in multipath fading or signal shadowing environments. Recent research has employed advanced ML-based approaches for jamming detection and classification. Only a small number of papers in the literature have proposed this approach; however, the results from this limited amount of work are promising. An important future research area would be to expand upon this work and continue maturing ML-based approaches to GPS jammer detection.

The topic of GPS spoofing detection has been extensively studied. Consequently, there is a large amount of research on this subject. The most common approach for GPS spoofing detection is ML-based, which has demonstrated strong performance. There is always room for improvement in any technical approach; however, this research topic has matured.

Despite the wide range of existing methods and approaches for positioning and navigation in GPS-denied environments, existing methods are either scenario- or environment-specific or have other significant limitations. There are many research opportunities for many of the individual alternate positioning and navigation approaches. Research into self-calibrating multi-IMU approaches is promising, but additional research is required to mature the concept, particularly for harsh environments and platform dynamics. Future research should address the security of using unauthenticated RF landmarks. Research is needed to reduce the computational complexity of visual methods of positioning and navigation. Furthermore, methods to improve the performance of visual methods in low-light and changing environmental conditions should be explored. Star tracker approaches should mature through future research. This research area is relatively dormant, but ongoing research is promising, and we believe it could represent an opportunity to provide a reliable means of navigation for many unmanned systems.

Currently, no existing approaches provide universally good performance with reasonably low complexity. Some promising techniques are emerging; however, those most promising techniques would be susceptible to many of the same threats faced by GPS. We have not yet converged on a mature technical solution to this problem due to the wide range of existing methods and approaches, all of which have strengths. Every existing method and approach work well in some situations but poorly in others; therefore, it is possible that there is no single best solution to this problem and that ensemble solutions will be required. From the literature, it appears that researchers, in general, have come to this realization. Most of the recent research papers focused on multi-sensor fusion approaches, where multiple sensor inputs were used jointly to develop position and navigation solutions. It is expected that this trend will continue. One limitation of the current multi-fusion approaches is that they are almost universally focused on two particular sensor data types and are not expandable or modular to incorporate new sensor types. A key future research area would be the development of a generalized multi-sensor fusion framework that could accommodate any sensor type possibly encountered in a plug-and-play fashion. This envisioned framework could operate with any subset of the total possible sensors without configuration or tailoring, which is vital because every UAS platform will likely have a slightly different hardware configuration with different sensors and processing capabilities. The standard approach to multi-sensor fusion is to input data from multiple orthogonal sensors into a Kalman filter to fuse the data and generate a state estimate of the system. The Kalman filter excels at fusing inputs that contain uncertainty due to noise; however, it struggles when a sensor returns anomalous data. Various sensors and approaches have strengths and weaknesses and may produce data that are not relevant in certain environments and conditions, leading to anomalous data that could negatively harm traditional data fusion approaches. Future multi-sensor fusion methods should consider the relevance of sensor data.

A key trend observed in the literature is that recent research across all aspects of detection and mitigation is adopting ML-based approaches, which is expected since ML-based approaches can yield very good results; however, ML-based approaches can be computationally expensive and may not be well-suited for smaller and low-cost UAS platforms. Research into applying sparse dataset training methods, such as zero-shot detection for visual methods, may prove useful.

Finally, based on the strengths and weaknesses of methods found in existing literature, the community has likely not yet converged on an optimal solution, and we must continue to further explore new detection and mitigation strategies against the threats faced by GPS.

## Figures and Tables

**Table 1:** High-level summary of the existing research on the topic of GPS security and alternate positioning.

Survey Paper	Topics Covered			Specific Topics		
	Attacks	Detection	Mitigations	GPS Spoofing	GPS Jamming	Alternative (Non-GNSS) Positioning and Navigation
[1]	X	X	X	X		
[2]	X		X		X	
[3]	X			X		
[4]	X	X	X	X		
[5]	X		X		X	
[6]	X		X		X	
[7]	X	X	X	X	X	
[8]	X	X	X	X		
[9]	X	X	X	X		
[10]	X	X	X	X		
[11]						X
[12]						X
[13]						X
[14]						X
[15]						X
[16]						X
[17]						X
[18]						X
[19]						X
[20]						X
[21]						X
[22]						X
[23]						X
[24]						X
[25]						X
[26]						X
Our paper	X	X	X	X	X	X

**Table 2:** Summary of alternate (non-GNSS) positioning and navigation survey papers.

Survey Paper	Type of Survey			Alternate Positioning and Navigation Methods Discussed in the Survey						
	Technical Approaches	Performance Analysis	Research Directions	RF-Based	Visual	Visual SLAM	Lidar-SLAM	Algorithm Evaluation	AI/ML Applications	IMU
[11]	X		X		X	X	X			X
[12]	X	X			X	X	X			
[13]	X	X			X	X			X	
[14]	X				X	X				
[15]	X	X						X		
[16]	X				X	X				
[17]	X					X				
[18]	X	X	X			X				
[19]	X	X				X				
[20]	X	X	X			X	X			
[21]	X						X			
[22]	X					X	X			X
[23]	X					X				X
[24]	X						X			
[25]	X	X				X			X	
[26]	X								X	
This paper	X	X	X	X	X	X	X	X	X	X

**Table 3:** Summary of GPS performance analysis.

Authors	Reference	Type of Results	Scenario/Conditions Considered	Factors Considered	General Conclusions of Accuracy
U. Engel	[34]	Theoretical	Various	Clock error, orbit error, refraction, multipath, code-tracking error	Position accuracy: 5–30 m
M. Rychlicki et al.	[35]	Experimental	Open stationary, open mobile, urban stationary	Variability across GPS receivers	HDOP: 0.7–1.2 VDOP: 0.9–1.6
J. Salas and M. Torroja	[36]	Experimental	Open stationary, open mobile	Variability across GPS receivers	Position accuracy: 0–4 m
M. Modsching et al.	[37]	Experimental	Urban stationery	Variability across GPS receivers	Position accuracy: <28 m for 95% of the time
P. Misra et al.	[38]	Theoretical, experimental	Various	Geometry, number of satellites, ranging errors, types of receiver signal processing and hardware	Position accuracy: 0.01–30 m
R. Conley	[39]	Theoretical	Various	Location on Earth, various error sources	Variable: centimeters to 10’s of meters
J. Spilker Jr.	[40]	Theoretical	Various	Various	Position accuracy: <10 m
D. Skournetou and E. Lohan	[41]	Theoretical	Open	Single vs. multi-frequency receivers	Ranging accuracy: 10–100 m
K. Merry and P. Bettinger	[42]	Experimental	Urban stationery	Multipath propagation	Position accuracy: 7–13 m
K. Chiang et al.	[43]	Theoretical, Experimental	Urban stationary, urban mobile	Multipath propagation	Position accuracy: <5 m
A. Hussain et al.	[28]	Theoretical	Urban stationary, urban mobile	Multipath propagation	N/A—Focus on detection and acquisition of GPS signals

N/A: Not Applicable.

**Table 4:** Summary of GPS degradation factors.

GPS Degradation Factor	Summary	Difficulty of Implementation	Required Expertise	Likelihood	Effect	Scope of Effect	Possible Ramification
Multipath Fading/Shadowing	Complex urban environment degrading GPS reception	N/A—natural condition	N/A—natural condition	High	Performance degradation or total GPS signal loss	Localized to urban centers	GPS-based navigation is not possible or causes crashes or impacts due to position error
Unintentional interference	Unintended emissions in GPS frequency bands	N/A—unintended action	N/A—unintended action	High	Performance degradation or total GPS signal loss	Localized to sources of interference	GPS-based navigation is not possible or causes crash or impact due to position error
Jamming	Intentional emissions in GPS frequency bands	Very low—can be implemented with low and no-cost commercial hardware and software	Low—basic SDR, RF hardware, and software development expertise or low-cost commercial jammer	High	Performance degradation or total GPS signal loss	Localized-to-wide area of effect	GPS-based navigation is not possible or causes crashes or impacts due to position error
Spoofing	Intentional broadcast of falsified GPS signal	Very low—low and no-cost commercial hardware and software	Low—basic SDR, RF hardware, and software expertise	High	GPS receiver reports an incorrect position	Localized-to-wide area of effect	Vehicle under spoofer control—could lead to loss of property or life

**Table 5:** Mean weekly number of different types of interference events from the measurement campaign presented in [44].

Location	AWGN Wideband	Narrowband Single-Tone	Chirp	CDMA	Other
Site 1	16.2	9.3	0.5	0.1	0.7
Site 4	37.3	4.8	1.2	0.8	0.5
Site 5	12.0	11.9	13.5	1.5	7.1
Site 7	12.9	43.1	2.8	1.1	1.8
Site 8	124.2	131.2	73.8	8.3	28.2
Site 9	10.0	3.7	3.5	0.5	11.0
Site 10	42.9	23.3	38.0	3.7	23.3

**Table 6:** Summary of low-cost GPS jammer implementations.

Authors	Reference	Difficulty of Implementation	Type of System	RF Hardware Platform	Signal Generation Environment
Farlik et al.	[48]	Low	Commercial	Commercial GPS Jammer	Commercial GPS Jammer
Saputro et al.	[50]	Low	SDR-based	BladeRF x40	GNU Radio
Ferreria et al.	[49]	Low	SDR-based	BladeRF x40	GNU Radio
Karpe and Kulkarni	[51]	Low	SDR-based	Unknown	GNU Radio
R. Ferreira et al.	[52]	Low	SDR-based	BladeRF x40	GNU Radio

**Table 7:** Summary of effectiveness of different GPS jamming strategies presented in [53].

Type of Jamming	Resulting BER (%)
Pulse Jamming	4–8%
CW Jamming	18%
Barrage Noise Jamming	14%
Swept PBN Jamming	2–4%

**Table 8:** Summary of low-cost GPS spoofer system implementations.

Authors	Reference	Difficulty of Implementation	Type of System	RF Hardware Platform	Signal Generation Environment
Satyanarayana et al.	[62]	Low	SDR	HackRF One	GPS-SDR-SIM GPS
Ueki et al.	[63]	Low	SDR	BladeRF x40	GPS-SDR-SIM GPS
Saputro et al.	[50]	Low	SDR	BladeRF X40	GPS-SDR-SIM GPS
Songala et al.	[64]	Low	SDR	HackRF One	GPS-SDR-SIM GPS
Karpe and Kulkarni	[51]	Low	SDR-based	Unknown	GNU Radio

**Table 9:** Summary of common approaches for GPS jamming detection.

Type of Approach	Underlying Concept	Strengths	Key Open Research Questions
Signal Statistics-based	Monitor received signal attributes and attribute changes in statistical properties to jammer	Simple to implement, based on easily observable parameters	How will these approaches work in complex propagation environments?
Antenna-based	Utilize antenna array to measure aspects of signal to discern between authentic signals and jammer signals	Ability to jointly detect and mitigate interference	Can antenna arrays be made sufficiently simple to be viable for small platforms?
Learning-based	Fuse attributes of GPS signal, jammer signal, and GPS receiver into predictive ML model	Among the best performing approaches in open literature	Can ML models be sufficiently optimized to run on small platforms with limited computational capability?

**Table 10:** Summary of most common approaches in the literature to GPS spoofing detection.

Type of Approach	General Idea	Strengths	Key Open Research Questions
ML-based	Use measurable features of GPS signal, spoofed signal, and GPS receiver to train an ML model for future predictions on future data based on those same features.	Based on easily observable parameters. Demonstrated good performance.	Can ML models be sufficiently optimized to run on small platforms with limited computational capability?
Antenna/DOA-based	Utilize antenna array to measure aspects of signal to discern between authentic signals and spoofed signals.	Based on easily observable parameters, few computational requirements. Demonstrated good performance.	Can antenna arrays be made sufficiently simple to be viable for small platforms?
Movement tracking-based	Use the movement history of the platform to identify anomalies and outliers in position estimates.	Simple to implement, few computational requirements. Demonstrated good performance.	How will these approaches work for complex flight paths?

**Table 11:** Summary of select ML-based approaches for GPS spoofing detection.

Authors	Reference	Chosen Features Summary	ML Model	Performance Metrics	Achieved Performance
A. Gasimova et al.	[73]	C/No, Various correlator values, Prompt Quadrature Component, Carrier Doppler, Pseudo-Range (PR), Receiver Time, Time of Week, Carrier Phase Cycles, SVN	Ensemble: Stacking	Accuracy Prob Detection Prob Misdetection Prob False Alarm	>95% >99% ~0.5% ~0.1%
C. Titouna and F. Abdelleselam	[74]	SVN, SNR, PR, Doppler Shift, Current Position, Previous Position, Neighbor Position (Swarm)	Bayesian Network	Precision Recall Area under ROC	>90% >85% 0.962
P. Jiang et al.	[75]	Speed, Direction	Recurrent Neural Network	Detection Rate False Alarm Rate	>85% <6%
S. Zuo et al.	[76]	SVN, PR, Doppler Shift, Carrier Phase Frequency Shift, SNR	Isolated Forest	Accuracy	>95%
M. Manesh et al.	[77]	SVN, Carrier Phase, PR, Doppler Shift, SNR	Neural Network	Accuracy Prob Detection Prob False Alarm	~100% ~100% ~0%
T. Khoei et al.	[78]	SVN, Doppler Shift, PR, Receiver Time, Carrier Phase Shift, Various Correlator values, Prompt In-Phase, Prompt Quadrature, Carrier Doppler, SNR	Ensemble: 10 ML models dynamically selected	Accuracy Prob Detection Prob False Alarm Prob Misdetection Processing Time	99.6% 98.9% 1.56% 1.09% 1.24%
M. Nayfeh et al.	[79]	Position, Time, Altitude, GPS speed, Type of GNSS fix, HDOP, VDOP, GPS Noise, Jamming State, Velocity, Number of Satellites, Heading, Timestamp	Decision Tree	Detection Rate Misdetection Rate False Alarm Rate	92% 13% 4%
G. Aissou et al.	[80]	PRN, DO, C/No, Others (Total of 11 Features)	Decision Tree (XGBoost)	Accuracy Prob Detection Prob Misdetection Prob False Alarm	95.5% 95.4% 4.6% 4.3%
S. Semanjski et al.	[81]	C/No, PR, Carrier Doppler, Others (Total of 11 Features)	SVM	Accuracy Prob Misdetection Prob False Alarm	97.8% 7.6% 1.5%
X. Wie et al.	[82]	Magnetometer X-Axis, Mean GPS Altitude, Mean Latitude (Total of 21 Features)	RF, XGBoost	Accuracy Precision Recall F1	99.69% 98.76–99.07% 99.38–99.69% 99.22%
X. Wie et al.	[83]	Latitude, Longitude, Altitude, Speed (Horizontal and Vertical), Roll, Pitch, Yaw, Roll Rate, Pitch Rate, Yaw Rate, Vertical Acceleration	SVM, KNN, RF, GBDT, DT, MLP, XGBoost	Accuracy Precision Recall Missing Mistake F1	97.70% 98.70% 96.76% 3.24% 1.32% 97.72%

**Table 12:** Summary of antenna-based approaches found in the literature for GPS jamming mitigation.

Author	Reference	Type of Proposed Approach	Antenna Technology	Measured Attributes
S. Ni et al.	[71]	Detection Algorithm Adaptation Algorithm	Generic array	Carrier Phase
N. Rezazadeh et al.	[101]	Antenna Design	Multimode microstrip	N/A
Y. Zheng et al.	[102]	Antenna Design	Planar array with annular ring array elements	N/A
V. Obi et al.	[103]	Antenna Design	Planar array with dipole array elements	N/A
L. Dan et al.	[104]	Adaptation Algorithm	Generic array	Delay estimation, C/A correlation
M. Jayaweera et al.	[88]	Detection Algorithm Adaptation Algorithm Antenna Design	Microstrip patch	Carrier Phase
B. Hao et al.	[105]	Antenna Design Adaptation Algorithm	Dual-polarized Ellipsoid array	Power, polarization mismatch

**Table 13:** Summary of signal processing-based approaches for GPS jamming mitigation.

Author	Reference	Technical Approach	Jamming Threats Addressed
Y. Chien	[106]	Adaptive Notch Filter (ANF)	CW interference
M. Abbasi et al.	[107]	ANF combined with neural network	CW interference
S. Kim et al.	[108]	Transversal Finite Impulse Response (FIR) Filter	Chirp jamming
S. Arif et al.	[68]	Complex Adaptive Notch Filter (CANF)	CW interference

**Table 14:** Summary of positioning approaches.

Positioning Method	Hardware Requirement	Advantages	Disadvantages	Level of Research Activity
GPS	GPS receiver	Small lightweight hardware. Low-cost hardware. Proven performance.	Vulnerable to spoofing and jamming attacks. Poor performance in multipath and shadowing environments.	Moderate—Some ongoing research on GPS performance.
Receiver-based GPS Performance Improvement	RF hardware (e.g., antenna)	Demonstrated ability to recover GPS performance in the presence of degradation.	Additional hardware or computational complexity.	Moderate—Continuing research in antenna-based and signal processing-based methods for jamming and spoofing suppression/rejection.
IMU	IMU	Good performance for short flight times. Successfully used to augment GPS when coverage is intermittent.	Accuracy decreases over time due to error accumulations.	High—Stand-alone IMU research is receiving moderate interest. Significant amount of research in multi-sensor IMU methods.
RF Landmark	RF receiver	High accuracy demonstrated, particularly in dense RF environments. Proliferation of commercial wireless infrastructure lends itself to wide applicability.	Some approaches require pre-placed signals along path of movement. Requires onboard database of known landmark locations. Performance dependent on environment and geometry.	Moderate—Various commercial communications infrastructures have been considered in the literature to serve as landmarks.
Visual Landmark	Camera	High accuracy has been demonstrated. Minimal hardware requirements.	Some approaches require pre-placed physical objects along path of movement. Requires onboard database of known landmark locations.	Moderate—Research often coupled with IMU-based methods. This area is more active within the context of Visual SLAM.
Star Tracker	Star tracker	Good performance in certain conditions.	Limited utility in daytime or when night sky obscured. Requires additional star tracker hardware. Can typically only provide accurate orientation.	Low—Limited research related to interference suppression and daytime operations.
Alternate GNSS	GNSS receiver	Low-cost, small hardware, often the same hardware as the GPS receiver.	Vulnerable to spoofing and DoS attacks. Poor performance in multipath and shadowing environments.	Low—Limited research on multi-GNSS performance.
Mega LEO Constellation	RF receiver	High accuracy has been demonstrated Mega-constellations less vulnerable to physical attacks.	Vulnerable to spoofing and DoS attacks. Additional hardware required. Many approaches require changes to existing constellations.	Moderate—Growing research area focused on using commercial LEO constellations for navigation.
LIDAR SLAM	LIDAR transceiver	Proven performance, has been in use for many years.	Expensive hardware. Computationally expensive. Typical application is regional mapping only.	Moderate—Mature research area. Perceived decline in research over time. Three-dimensional is more researched than two-dimensional.
Visual SLAM	Camera	Proven performance, has been in use for many years. Minimal sensor hardware requirements.	Computationally expensive. Typical application is regional mapping only. Poor performance in low-light.	High—Significant research in the space of Visual-SLAM. Focus on multi-sensor fusion and IMU-hybrid methods.
Generalized Vision	Camera	Good performance possible. Minimal additional hardware requirements.	Computationally expensive.	Moderate—Less active research area compared with Visual-SLAM. Much research in the context of hybrid IMU approaches.
